# Intra-host genomic diversity and integration landscape of human tissue-resident DNA virome

**DOI:** 10.1093/nar/gkae871

**Published:** 2024-10-22

**Authors:** Lari Pyöriä, Diogo Pratas, Mari Toppinen, Peter Simmonds, Klaus Hedman, Antti Sajantila, Maria F Perdomo

**Affiliations:** Department of Virology, University of Helsinki and Helsinki University Hospital, Haartmaninkatu 3, P.O. Box 21, FI-00014, Helsinki, Finland; Department of Virology, University of Helsinki and Helsinki University Hospital, Haartmaninkatu 3, P.O. Box 21, FI-00014, Helsinki, Finland; IEETA, Institute of Electronics and Informatics Engineering of Aveiro, and LASI, Intelligent Systems Associate Laboratory, University of Aveiro, Campus Universitário de Santiago, 3810-193, Aveiro, Portugal; Department of Electronics, Telecommunications and Informatics, University of Aveiro, Campus Universitário de Santiago, 3810-193, Aveiro, Portugal; Department of Forensic Medicine, University of Helsinki, Haartmaninkatu 3, P.O. Box 21, FI-00014, Helsinki, Finland; Nuffield Department of Medicine, University of Oxford, Peter Medawar Building, South Parks Road, OX1 3SY, Oxford, UK; Department of Virology, University of Helsinki and Helsinki University Hospital, Haartmaninkatu 3, P.O. Box 21, FI-00014, Helsinki, Finland; Department of Forensic Medicine, University of Helsinki, Haartmaninkatu 3, P.O. Box 21, FI-00014, Helsinki, Finland; Forensic Medicine Unit, Finnish Institute for Health and Welfare, Mannerheimintie 166 A, P.O. Box 30, FI-00271, Helsinki, Finland; Department of Virology, University of Helsinki and Helsinki University Hospital, Haartmaninkatu 3, P.O. Box 21, FI-00014, Helsinki, Finland

## Abstract

The viral intra-host genetic diversities and interactions with the human genome during decades of persistence remain poorly characterized. In this study, we analyzed the variability and integration sites of persisting viruses in nine organs from thirteen individuals who died suddenly from non-viral causes. The viruses studied included parvovirus B19, six herpesviruses, Merkel cell (MCPyV) and JC polyomaviruses, totaling 127 genomes. The viral sequences across organs were remarkably conserved within each individual, suggesting that persistence stems from single dominant strains. This indicates that intra-host viral evolution, thus far inferred primarily from immunocompromised patients, is likely overestimated in healthy subjects. Indeed, we detected increased viral subpopulations in two individuals with putative reactivations, suggesting that replication status influences diversity. Furthermore, we identified asymmetrical mutation patterns reflecting selective pressures exerted by the host. Strikingly, our analysis revealed non-clonal viral integrations even in individuals without cancer. These included MCPyV integrations and truncations resembling clonally expanded variants in Merkel cell carcinomas, as well as novel junctions between herpesvirus 6B and mitochondrial sequences, the significance of which remains to be evaluated. Our work systematically characterizes the genomic landscape of the tissue-resident virome, highlighting potential deviations occurring during disease.

## Graphical Abstract

**Graphical Abstract:**
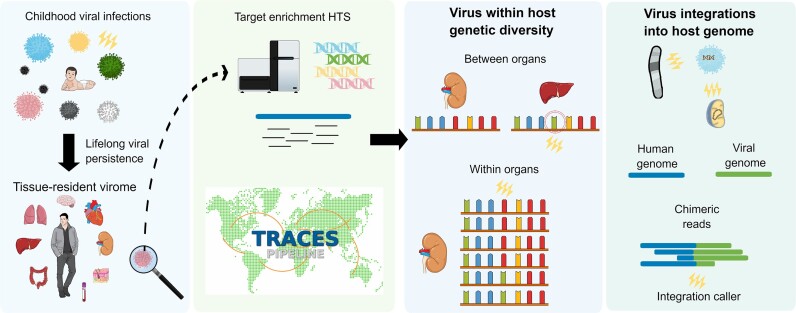

## Introduction

Advances in high-throughput sequencing (HTS) have enabled the identification of viral communities in human samples in unprecedented detail (1–3). This technology has opened new avenues for the exploration of viral diversity, evolutionary dynamics and even the occurrence of integrations in the host genome (4). However, the genetic composition of the tissue-resident virome of humans has remained obscure primarily because of shallow sequencing. This has hindered thorough characterization of viral genomes and detailed analysis of viral subpopulations (3,5,6). Such investigations could, however, reveal novel insights into pathogenesis, tissue tropism, drug resistance or the host selective pressure driving viral evolution (7–13). Since persisting viruses typically have low viral loads, the intra-host diversities have only been examined for selected species, mainly in the context of primary infections or reactivations, and mostly through bodily fluids or blood samples. Thus, sequence variations affecting tissue tropism and the within-host evolution during latency have largely remained unexplored (14–21).

We recently uncovered the anatomical distribution of human DNA viruses persisting in nine organs of 31 recently deceased individuals (3). Using hybrid capture sequencing, we provided unparalleled genomic resolution for human herpesviruses (HHVs 1, 3, 4, 5, 6B and 7), JC and Merkel cell polyomaviruses (JCPyV and MCPyV), and human parvovirus B19 (B19V).

In the present study, we performed in-depth analyses of these viral genomes to assess the intra-host and within-organ diversities. To this end, we employed two approaches: first, we analyzed the inter-tissue variabilities by comparing the viral consensus sequences reconstructed from different organs in an individual; second, we determined the viral genome heterogeneity within each tissue type by conducting minor variant (MV) analysis (i.e. of non-consensus variants) (16,18,22).

In addition, we explored viral integrations into the human genome by assessing chimeric (viral-host) sequencing reads. Given the technical challenges of identifying unknown junctions prior to the advent of HTS, the frequency of integrations, especially within healthy tissues, has remained largely unknown. While these insertions have been primarily associated with the onset of human cancers (e.g. with human papillomaviruses [HPVs], hepatitis B virus [HBV] and MCPyV) (23–26), recent findings have revealed multiple integration sites across human chromosomes, not only in tumors but also in adjacent tissues (27–30). Such integrations could unveil unexpected associations with the onset of diseases and shed light onto viral mechanisms of persistence within tissues.

This study provides new insights into the genomic diversities of DNA viruses residing within an individual and reveals an intricate landscape of viral integrations into the human genome.

## Materials and methods

### Cohort

The raw paired-end virus sequencing data were obtained from a total of 96 samples of thirteen individuals (average age 71), originating from a previous study conducted by us. In it we examined the composition, distribution and intra-host phylogeny of the eukaryotic DNA virome across nine different tissues: brain, colon, heart, liver, lung, kidney, skin, whole blood and plucked hair (3). Out of these 13 individuals, eleven exhibited average persistent virus species and copy numbers (baseline levels). In contrast, the remaining two had putative reactivations of HSV-1, HCMV, JCPyV and EBV or VZV and EBV based on the quantities and systemic distribution (Supplementary Table S1). These two individuals suffered from malignant conditions (metastatic pulmonary carcinoma; relapsed mantle cell lymphoma [MCL]). The latter had been treated with acyclovir for facial herpes zoster five weeks before death.

In the individuals with baseline virome, the causes of death were either trauma (36%), heart disease (45%) or consequences of alcohol abuse (18%). For most, medico-legal autopsy also showed evidence of coronary artery disease and other atherosclerotic changes as well as hepatic steatosis which are common findings in this age demographic.

### Viral genome reconstruction

Binary Alignment Maps for each virus were constructed using TRACESPipe v.1.1, with read duplicates removed. The consensus sequences were built based on reference-based viral alignments and *de novo* assemblies, as described (3,31).

### Intra-host variation between tissues

The consensus sequences generated were aligned and compared in Geneious Prime (v.2022.2.2). Sequences reconstructed from at least two tissues of an individual were included in the analysis (coverage range: 47–100%, median: 93%). The dissimilarities among consensus sequences were reviewed using the aligned reads in Integrative Genomics Viewer (IGV, v.2.15). Only variations supported by at least two sequence reads with minimum Phred quality score of 25 were included.

Repeat regions, based on annotation from the NCBI GenBank, and extremely low complexity regions, determined with AlcoR (32), were excluded from the analysis. An exception were the inverted terminal repeats (ITRs) of B19V (∼383 bp length), which were analyzed by visual inspection with IGV (33).

### Minor variant analysis

MV calling was conducted using iVar (v1.3.1) (34), applying a significance threshold of *P*< 0.05 and considering alternative nucleotide frequencies from 3% to 50%. A minimum of four supporting reads with Phred quality score of at least 20 were used as cut-off. As with the consensus sequence comparisons, repeat regions were excluded from the MV analyses, except for the B19V ITRs.

### Minor variant indels in short microsatellites

MV indels occurring in short microsatellite areas were confirmed, in addition to iVar, with another variant caller LoFreq (v2.1.5) (35). However, variant callers may be prone to miscalling indels within microsatellites, as these areas are more susceptible to sequencing artifacts, e.g., due to polymerase slippage (PCR stutter). To this end, we applied GangSTR (v2.5) (36), a genotyper for tandem repeat expansions from paired-end short-read sequencing data, to scrutinize potential heterogenous repeats identified with variant callers. This program, among other variables, takes into consideration PCR stutter background. Microsatellite indels were included in the results only if GangSTR supported two versions of a repeat within the sample. Additionally, sequences extending 50 bp on both sides of identified MV microsatellites were subject to BLAST analysis to exclude matches from other organisms.

### B19V hairpin analysis

The B19V ITRs, were inspected using IGV. Each read with a detected indel was subjected to BLAST analysis to exclude misalignment artifacts. The self-folding was predicted using Mfold web server (37) with default settings, and a temperature of 37°C.

### Integration analysis

Potential virus–host integration junctions were investigated with a repeat-aware integration caller, SurVirus, configured with default settings. This caller was selected for its conservative approach, offering greater specificity compared to many alternatives, as demonstrated in benchmark tests with datasets of known integrations (38). As a human reference, the genomes CRCh38/hg38 and the T2T-CHM13v2.0/hs1 were used (39,40). Reads suggestive of integration were further examined to validate partial matches to human and viral genomes using BLAST (41). The locations of these junctions within the host were confirmed by realigning the sequences adjacent to the breakpoints with BLAT (using reference T2T-CHM13v2.0/hs1) (42). The host regions adjacent to the junctions were investigated with a genome browser (43).

The virus-host split reads, as well as the viral and host sequences adjacent to the junction, were aligned using Geneious Prime to explore potential mechanisms of integration, such as microhomology-mediated end-joining (MMEJ) (44,45). This process involves the insertion of viral DNA into the host genome through short sequences that exhibit high homology.

### Detection of large structural variations

DELLY call (Galaxy Europe version 0.9.1 + galaxy1) was used with default settings (46,47) to investigate large truncating deletions within MCPyV, JCPyV, HPyV6 and B19V genomes. The reads supporting the deletions were visually verified with IGV.

### Statistical analysis

The intra-host diversities of viral consensus sequences between organs of each individual were assessed by the mean number of mutations (substitutions, insertions and deletions) between consensus sequences per site (mutations/site) and nucleotide diversity ($\pi = \frac{D}{{L \cdot \frac{{n( {n - 1} )}}{2}}}$, where *D* is total number of differences in multiple sequence alignment, *L* is the length of genome/coding sequence (CDS) area/non-CDS area, and *n* number of aligned sequences). The intra-sample diversities were evaluated by the mean number of MV positions per site (MVs/site) and the mean site-wise Shannon index *H* = −∑ (*p*_i_⋅log2 (*p*_i_)), where *p* is the frequency of alternative nucleotides in each genome position. The metrics were compared between CDS and non-CDS within each virus species and between whole genomes of all different viruses using permutations test of means with 10 000 permutations. Bonferroni correction was utilized to calculate *P*-values with multiple comparisons. Two-sided binomial test was applied to assess the asymmetry of observed transition mutations. Chi-square test was used to compare expected versus observed number of integrations within genes or repeat regions. All statistical analyses were conducted using R (v.4.2.0), and *P*< 0.05 was considered statistically significant.

## Results

Viral genomic analyses were conducted from a total of 127 genomes, including parvovirus B19 (B19V), MCPyV, JCPyV and HHVs 1, 3, 4, 5, 6B, and 7, reconstructed from multiple organs of 13 individuals (Supplementary Table S2) (3).

For the inter-organ analysis, we included sequences that were present in at least two organs of the same individual, amounting to 110 consensus sequences. These sequences were derived from genomes with an average breadth coverage of 86% (range 47–100%) and depth of 44X (range 1–12227X). We searched for both consensus single nucleotide variations (cSNVs) and insertion/deletions (indels) between sequences. Additionally, we calculated the mean numbers of mutations per site (mutations/site, *M*) and average per-site nucleotide diversity (π) for the (i) whole genome, (ii) CDSs and (iii) non-CDSs.

**Figure 1.**
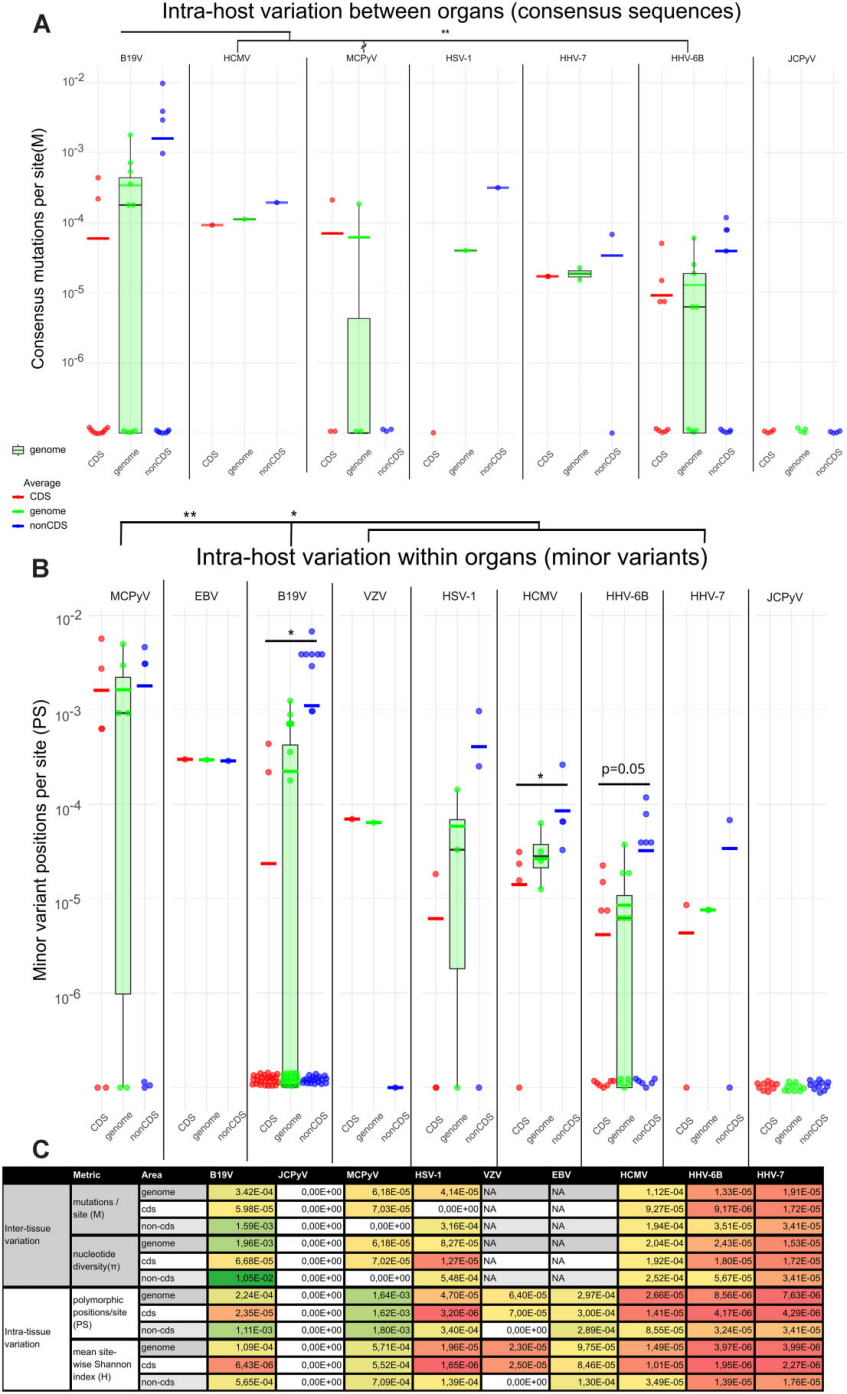
Intra-host genetic diversity of persistent DNA viruses in tissues. (**A**) Number of consensus mutations per site (M) when comparing organs within an individual. Each datapoint represents intra-host diversity in one individual. (**B**) Number of MVs (minor variant positions per site, PS), with each datapoint representing one sample. Different viruses are divided into separate panels, each containing a separate plot for CDS (red), whole genome (green) and non-CDS (blue). Horizontal lines illustrate the mean values. For the whole genome, a boxplot is shown. Datapoints at the bottom represent zero-diversity. CDS and non-CDS values within each virus species were compared with a permutation test of the means, with significant results shown in the figure. Whole genome diversity values were compared between each virus with the permutations test of the means, with Bonferroni correction for multiple comparisons. The brackets indicate a comparison of B19V or MCPyV to herpesviruses as a single group. *P< 0.05, **P< 0.01. Alternative metrics for inter-organ diversity (nucleotide diversity) and intra-organ diversity (mean site-wise Shannon index) are shown in Supplementary Figure S1. (**C**) Table with exact values of all the metrics of diversity (mutation/site, nucleotide diversity, polymorphic positions/site and mean site-wise Shannon index). Higher values are in shades of green and lower in yellow and red. B19V, human parvovirus B19; EBV, Epstein-Barr virus; HCMV, human cytomegalovirus; HHV-6B, human herpesvirus 6B, HHV-7, human herpesvirus 7; HSV-1, herpes simplex virus 1; JCPyV, JC polyomavirus; MCPyV, Merkel cell polyomavirus; VZV, varicella-zoster virus.

Based on both *M* and π, the whole genome inter-tissue diversity was higher for B19V (*M* = 3.4 × 10^−4^ mutations/site and π = 2.0 × 10^−3^) than for any of the HHVs (average of the family, *M* = 2.4 × 10^−5^ mutations/site and π = 4.2 × 10^−5^; permutation test for means, *P*= 0.007 and *P*= 0.02, respectively). With most virus species, non-CDS exhibited higher diversity compared to CDS (Figure 1A and C; Table 1; Supplementary Figure S1A and Supplementary Dataset S1).

**Table 1.** Statistics of inter-organ consensus differences and intra-organ MVs

Inter-organ consensus differences	B19V	MCPyV	HSV-1	HCMV	HHV-6B	HHV-7	Intra-organ MVs	B19V	MCPyV	HSV-1	VZV	EBV	HCMV	HHV-6B	HHV-7
Individuals	11	3	1	1	8	2									
Identical consensus sequences	5	2	0	0	3	0	**Genomes analyzed**	28	6	3	1	1	4	11	4
Samples	60	7	3	4	21	4	**Genomes without MVs**	19	2	1	0	0	0	5	2
No. of mutations	21	1	5	18	19	5	**No. of MVs**	35	53	12	7	51	19	15	2
CDS	3	1	0	12	11	5	**CDS**	3	46	1	7	37	7	7	1
Non-CDS	18	0	5	6	8	0	**Non-CDS**	32	7	11	0	14	12	8	1
cSNV	11	1	0	10	10	4	**iSNV**	7	50	11	4	47	9	8	2
Synonymous	3	1	0	4	3	0	**Synonymous**	0	15	0	0	13	2	1	0
Non-synonymous	0	0	0	4	4	4	**Non-synonymous**	3	28	1	3	22	2	4	1
Indel	10	0	5	9	9	1	**Indel**	28	3	1	3	4	10	7	0
Microsatellite indel	0	0	4	4	7	1	**Microsatellite indel**	0	0	0	1	3	1	7	0
Frameshift	0	0	0	0	3	1	**Frameshift**	0	1	0	3	0	0	0	0
Premature stop	0	0	0	0	0	0	**premat. stop**	0	0	0	1	0	0	0	0
**Genes	NS1(2) VP	VP2		UL112(3) UL44(2) UL33 UL36 UL80 UL95 UL115 UL116 US13	U21(2) U47(2) U13 U29 U59 U66 U79 U86 B5	U57(3) U31 U48	**Genes**	VP(2) NS1	VP2(23) LT(19) VP1(3) ST	US6	ORF6(2) ORF5 ORF12 ORF18 ORF30 ORF54	EBNA-2(5) EBNA-1(4) BBLF2/BBLF3 (3) LMP-1(3) BPLF1(2) BMRF2(2) EBNA-3C(2) BBRF1/2(2) BCLF1(2) BALF5(2) BNRF1 BHLF1 EBNA-3A BZLF1 BDLF3 BDLF2 BTRF1 BXLF1 BILF1 BALF2	UL42(5) UL116 UL122	U47(3) U86(3) U51	U30

*Indicates in which genes variations were seen with n.o of variations within brackets. B19V, human parvovirus B19; EBV, Epstein-Barr virus; HCMV, human cytomegalovirus; HHV-6B, human herpesvirus 6B, HHV-7, human herpesvirus 7; HSV-1, herpes simplex virus 1; JCPyV, JC polyomavirus; MCPyV, Merkel cell polyomavirus; VZV, varicella-zoster virus.

In addition to consensus sequences that represent the most commonly occurring nucleotide at each position, we investigated the presence of MVs within viral genomes. This pertains to positions where, in addition to the consensus nucleotide, at least one alternative nucleotide (s) exists, at a frequency of 3–50%, representing either intra-sample single nucleotide variations (iSNVs) or indels. We furthermore assessed how MV positions correlated to variations in the consensus sequences, determining the percentage of instances in which mutations at the consensus-level were also accompanied by MVs at the same position. Since our data lacked haplotype reconstruction, we quantified the intra-host genetic diversity using MVs by calculating the number of polymorphic sites per sequence length (taking into account richness; MVs per site, PS) and the mean site-wise Shannon entropy (which considers both richness and evenness; *H*) across the whole genome, CDS and non-CDS separately. To further understand the distribution of minor allele frequencies, we analyzed the site frequency spectrum (Supplementary Figure S2).

Based on the MV analysis, the whole genome intra-sample heterogeneity was higher for MCPyV (PS = 1.6 × 10^−3^ MVs/site, *H* = 5.7 × 10^−4^) and B19V (PS = 2.2 × 10^−4^ MVs/site, *H* = 1.1 × 10^−4^) than for HHVs (PS = 3.5 × 10^−5^ MVs/site, *H* = 1.3 × 10^−5^; permutation test for means, *P*< 0.001 for both metrics in MCPyV versus HHVs; and *P*= 0.02 for both metrics in B19V versus HHVs;). The intra-sample diversity was higher in non-CDS than in protein coding regions for most viruses, except for MCPyV, EBV and VZV, which exhibited higher or close to equal intra-host variability in CDS (Figure 1B and C; Table 1; Supplementary Figure S1B and Supplementary Dataset S2).

From herpesviruses, 58% (51/88) of mutations in CDS observed either between organs or within samples were found in genes encoding products targeted by virus-specific CD4 + or CD8 + T cells, indicating a possible selection pressure from host adaptive immunity (Table 1 and Supplementary Text S1-S5) (48–54).

### Parvovirus B19 genotype 2 shows higher within-host genetic diversity within inverted terminal repeats

**Figure 2.**
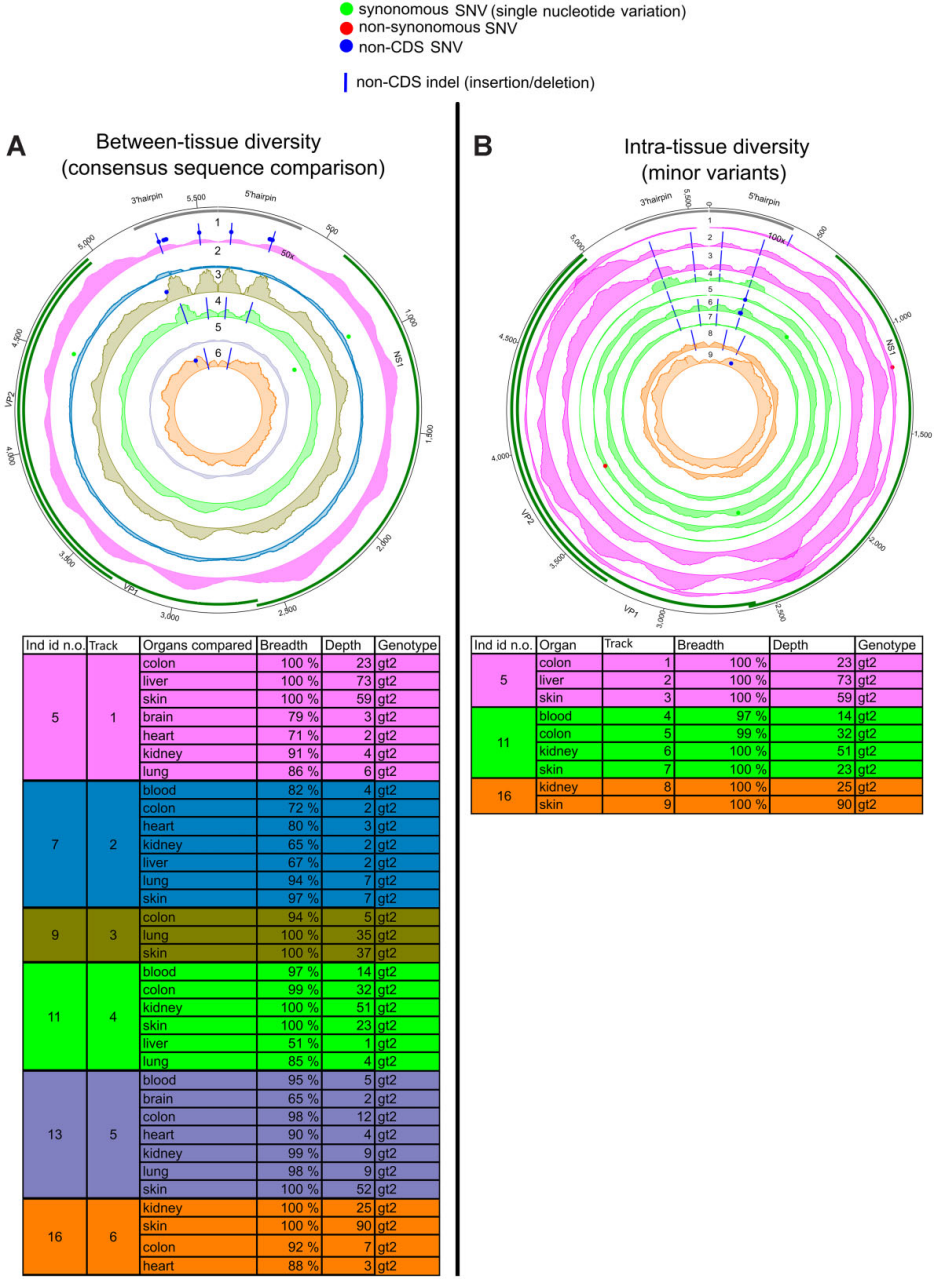
Intra-host variation of parvovirus B19. (**A**) Comparison of viral consensus sequences between different organs within an individual. Each circos plot track (numbered from 1 to 6) represents diversity in one individual, and the area illustrates an average depth coverage of the virus genomes (scale from 0 to 50×). (**B**) MV positions in each B19V genome. Each track (numbered from 1 to 9) represents one sample with unique individual color coding concordant with (A). The areas within tracks illustrate the depth coverage of the viral genome (scale from 0 to 100×). Both circos figures are plotted against reference genome AB550331.1. The black bars in the outer edge represent ITRs and the green bars genes. SNVs are marked with red (non-synonymous), green (synonymous) and blue (non-coding region) dots and indels with blue lines. Details of the samples are shown in both respective tables.

We compared B19V consensus sequences from 11 individuals, in altogether 60 samples (average breadth coverage 89% and depth 56×). Eight individuals harbored genotype 2 (gt2) and three, gt1. Strikingly, we found mutations exclusively in B19V gt2, detected across six individuals (Figure 2A; Table 1; Supplementary Dataset S1).

The inter-tissue diversity of B19V was the highest among all viruses studied, particularly within non-CDS (both *M* and π, *P*= 0.09). The majority of mutations were indels within ITRs (see section Indels in parvovirus B19 genotype 2 ITRs impact self-folding) (Figures 1A,C and 2A; Table 1; Supplementary Figure S1A). Consensus differences in CDS (VP and NS1 genes) were observed only in two individuals.

The highest frequency of consensus variations was found in the colon (48%), followed by the lung (24%), brain and skin (19%), liver and kidney (10%) and heart (5%).

Similarly to the consensus-level, MVs were identified only in individuals with gt2, in samples with over 97% breadth and 14× depth coverage. Specifically, we identified MVs in 9 out of 28 tissue samples from three individuals, from whom mostly full-length sequences were recovered (112×). We observed more MVs in non-CDS compared to CDS (*P*= 0.001 and *P*= 0.008 for PS and H, respectively, permutation of means) (Figure 1B and C; Table 1; Supplementary Figure S1B). Among the MVs, only two instances of iSNV were found in the coding regions, both from colon samples. Analogously to the consensus analysis, the majority of MVs were indels, and located within the ITRs (Figure 2B; Table 1; Supplementary Dataset S2). The mean frequency of alternative nucleotides at MV positions was 33% (Supplementary Figure S2A) and 77% of MV positions were shared at least between two samples.

We discovered that 48% of the locations in which B19V consensus sequences varied among organs exhibited a switch between major and MVs. This indicates potential fluctuation in the prevalence of subpopulations across different organs.

Notably, B19V cSNVs and iSNVs exhibited a marked asymmetry toward C to T and G to A mutations (59% of total SNVs and 91% of transitions) (*P*= 0.22 and *P*= 0.06, respectively) (Supplementary Figure S3A).

### Indels in parvovirus B19 genotype 2 inverted terminal repeats impact self-folding

We identified four novel 15-nt insertions within the ITRs of B19V gt2 genomes from two individuals (Supplementary Figure S4A). Both the 5′ and 3′ hairpins exhibited two complementary insertions. In one individual, the reads containing these insertions constituted a major variant in the kidney (63% of reads), while in the skin, colon and blood, they represented MVs (30–40% of reads). These insertions were absent in the liver and lung, although this can be due to lower depth coverage (1× and 4×, respectively). Similarly, in another individual, these insertions were a major variant in the skin and liver (∼60% of reads), but were absent in the brain, heart, kidney and lung.

The insertion pairs were 210 nt apart, making it challenging to ascertain whether these insertions consistently occurred as complementary pairs, or could exist independently. Folding analysis revealed that the presence of both insertions in a pair led to more stable self-folding of the ITRs, while the presence of only one insertion would hinder complementarity, resulting in less stable folding (Supplementary Figure S4B).

In addition to insertions, we identified two novel 12 or 13nt deletions within B19V gt2 in one individual’s kidney. The reads in this sample had either a 12 nt deletion located at positions 185 of the 5′ end and 5393 of the 3′ end (5′ CCGCTTGTCTTA; 3′ TAAGACAAGCGG) in approximately 54% of the reads or a 13nt deletion at positions 168 of the 5′ end and 5409 of 3′ end (5′ TAAGATCAAGCGG; 3′ CCGCTTGATCTTA), in ∼46% of the reads with respect to reference AB550331.1. These deletions were not detected in the skin or heart. Folding analysis showed that both deletions introduced mismatches in the self-folding process, resulting in thermodynamically less stable structures (Supplementary Figure S4B).

Furthermore, in one B19V gt2 positive individual, we detected a 9 nt deletion at positions 278 of the 5′ end (AAATGACGT) and 5303 of the 3′end (ACGTCATTT). This deletion was consistently present in all reads and tissues with at least 1× coverage in those regions (*n* = 3, max coverage depth 15× in this region). This deletion resulted also in a thermodynamically less stable structure compared to the reference ITR (AB550331.1), (Supplementary Figure S4B).

### Merkel cell, unlike JC polyomavirus, exhibits intra-sample diversity

**Figure 3.**
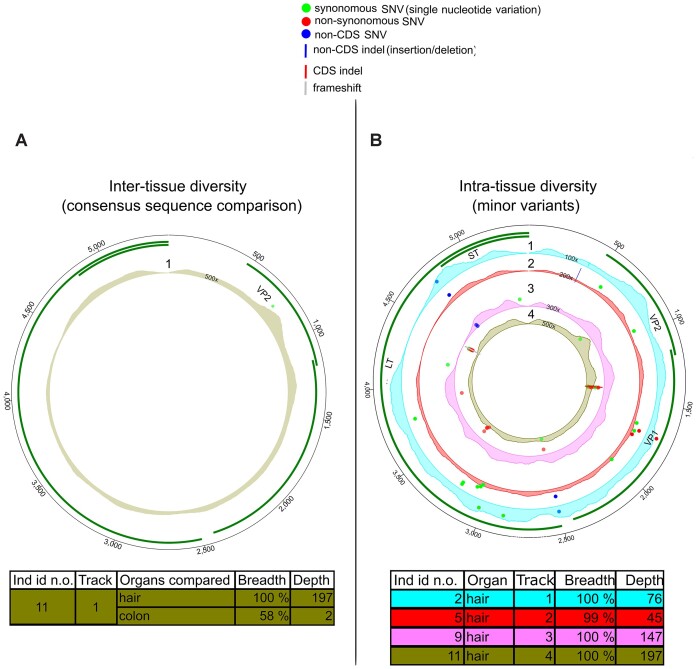
Intra-host variation of MCPyV. (**A**) Comparison of viral consensus sequences between hair and colon of a single individual. The area illustrates an average depth coverage of the virus genomes (scale from 0 to 500×). (**B**) MV positions in each MCPyV genome from plucked hair samples. Each track (numbered from 1 to 4) represents one sample with unique individual color coding concordant with (A). The areas within tracks illustrate the depth coverage of the viral genome (scale from 0 to 100–500×). Both circos figures are plotted against reference genome HM011544.1. Green bars in the outer edge represent genes. SNVs are marked with red (non-synonymous), green (synonymous) and blue (non-coding region) dots and indels with blue (non-CDS), red (CDS) or gray (frameshift) lines. Details of the samples are shown in the corresponding tables.

We conducted a comparative analysis of the inter-tissue variation of MCPyV among three individuals. In one, we observed a synonymous cSNV in the VP2 region, consisting of a C to T mutation in a colon sample (position 755 on reference HM011544.1) (Figure 3A; Table 1; Supplementary Dataset S1). In this identical position in hair samples, 29% of the nucleotides were T and 71% C, whereas in the colon, 83% were T and 13% C. These findings suggest that the change reflected in the consensus sequence resulted from a shift between major and minor nucleotides.

We identified MCPyV MVs in the plucked hairs of four out of six individuals (whole genome sequences, average 239× depth coverage) (Figure 3B, and Table 1; Supplementary Dataset S2). PS and H had similar values in both non-CDS and CDS (*P*= 0.95 and 0.75, respectively, permutation of means, Figure 1B and C; Supplementary Figure S1B).

MCPyV exhibited a higher number of MV positions per site than any other virus in the cohort (Figure 1B and C; Supplementary Figure S1B). Furthermore, nearly all MVs in MCPyV were iSNVs instead of indels (Figure 3B and Table 1; Supplementary Text S1).

In our analysis, JCPyV showed no intra-host genetic diversity at the consensus-level, based on four individuals. Likewise, MVs were not observed in any of the ten JCPyV genomes obtained from the kidney, liver, lung, whole blood, colon or plucked hair (average breadth coverage 93% and depth 27X, range 2–140×). This was also the case for the single human polyomavirus 6 genome reconstructed (97%, 8×) from a plucked hair sample.

### Roseolaviruses, human herpesvirus 6B and 7, exhibit low diversity

**Figure 4.**
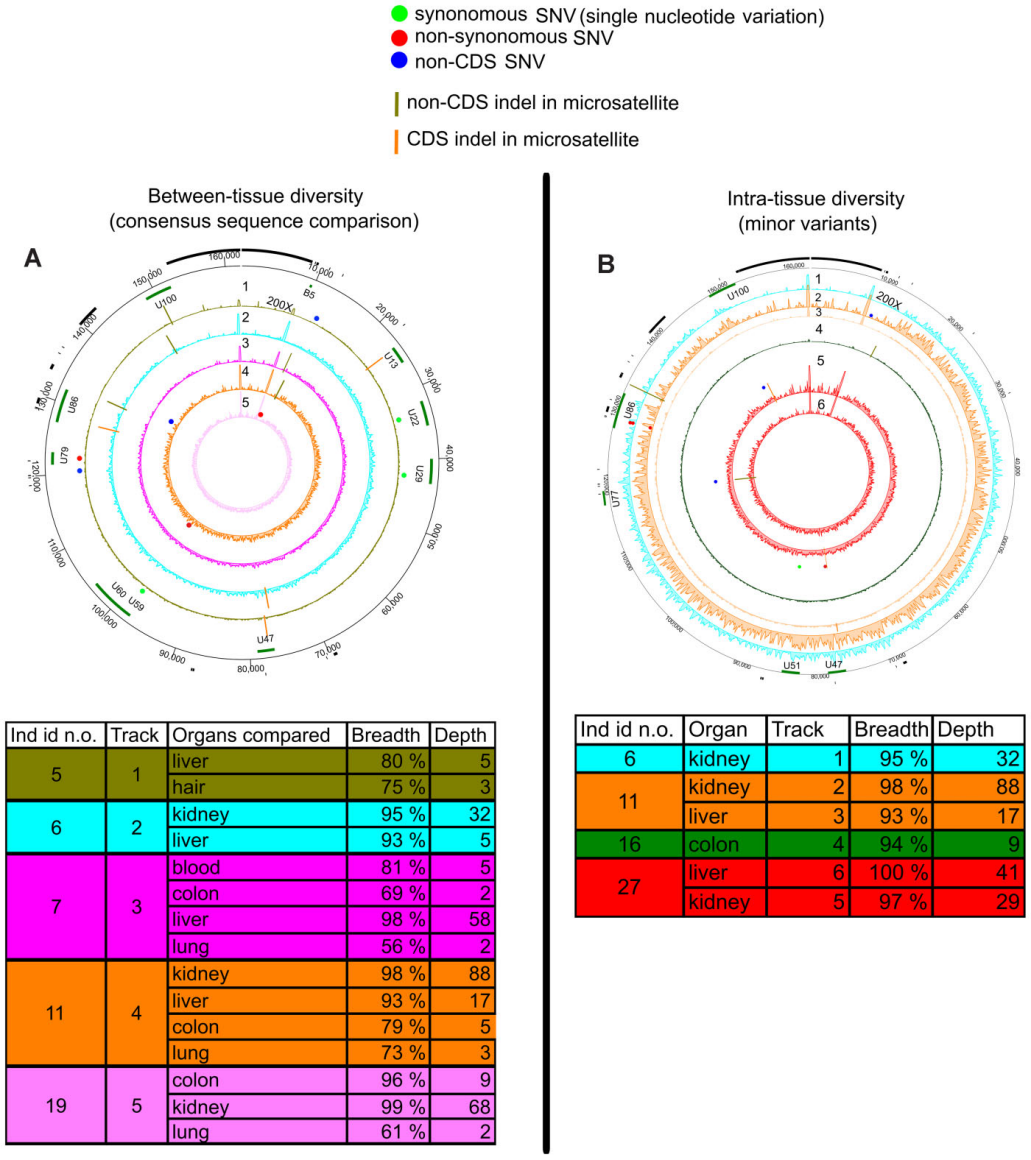
Intra-host variation of HHV-6B. (**A**) Comparison of viral consensus sequences between different organs within an individual. Each circos plot track (numbered from 1 to 5) represents diversity in one individual, and the area illustrates an average depth coverage of the virus genomes (scale from 0 to 200×). (**B**) MV positions in each HHV-6B genome. Each track (numbered from 1 to 6) represents one sample with unique individual color coding concordant with (A). The areas within tracks illustrate the depth coverage of the viral genome (scale from 0 to 200×). Both circos figures are plotted against reference genome MH698401.1. The black bars in the outer edge represent repeat regions masked from the analysis and the green bars genes. SNVs are marked with red (non-synonymous), green (synonymous) and blue (non-coding region) dots and indels with vertical lines. Details of the samples are shown in both respective tables.

We conducted a comparative analysis of HHV-6B genomes from eight individuals, predominantly across the colon, liver and kidney. In total, five individuals exhibited differences in their HHV-6B consensus sequences across different tissues (Figure 4A and Table 1; Supplementary Text S2 and Supplementary Dataset S1). The inter-organ variation rate among HHV-6B consensus sequences was slightly higher in non-CDS than in CDS, although not statistically significant (*P*= 0.14 and 0.33 for *M* and π, respectively, permutation test of means) (Figure 1A and C; Supplementary Figure S1A).

HHV-6B MVs were detected only in samples with minimum of 93% breadth and 10× depth coverages. From these, we identified MVs in 6/11 samples (average breadth and depth coverage: 96%, 36×). PS and H were higher in non-CDS vs. CDS (PS, *P*= 0.05; H, *P*= 0.03; permutation test of means) (Figure 1B and C, and Figure 4B; Supplementary Figure S1B, Supplementary Text S2Supplementary Dataset S2).

Both HHV-6B and HHV-7 exhibited lower intra-host diversity across all four metrics compared to other viruses studied (apart from JCPyV which showed no identifiable intra-host variability) (Figure 1; Supplementary Figures S1 and S5, and Supplementary Text S3).

### Higher herpesvirus diversities evidenced in malignancy

Two individuals (72 and 77 years old) within our cohort had been diagnosed with terminal cancers: one with stage IV relapsed MCL and another with terminal metastatic pulmonary carcinoma (PC), (Supplementary Table S1). Their core tissue virome differed from the rest of the cohort (3) with regard to copy numbers (up to 900 000 copies per million cells) and widespread prevalence of EBV and VZV (MCL) or HSV-1, HCMV and JCPyV (PC), suggestive of reactivations.

**Figure 5.**
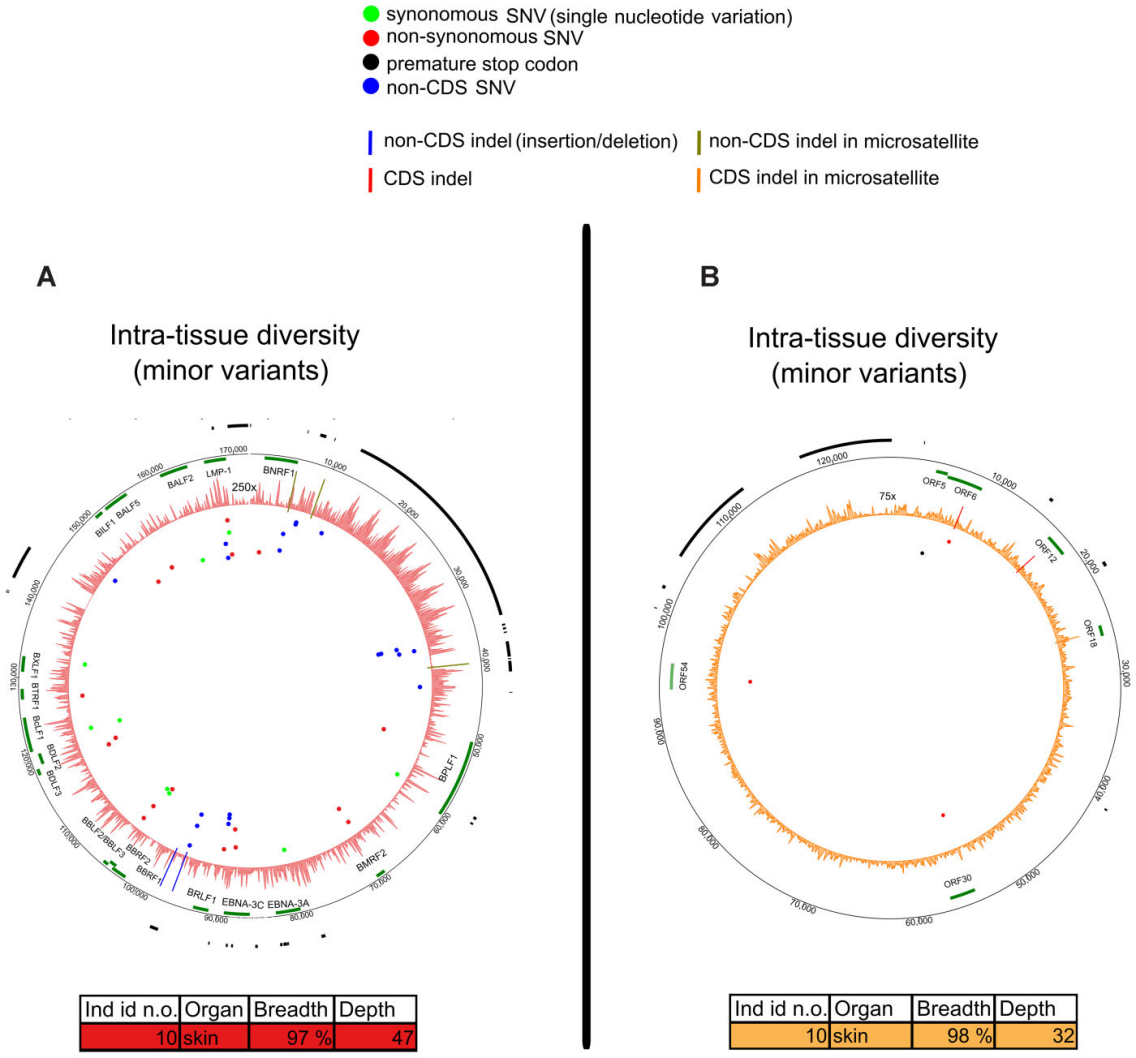
Intra-host diversity of EBV and VZV reconstructed from a skin sample of individual with relapsed MCL. MV positions in (**A**) EBV genome and in (**B**) VZV genome. Each track represents one sample, and the areas within tracks illustrate the depth coverage of the viral genome (scale from 0 to 250× or 0 to 75×). Circos figures are plotted against reference genome MG298924.1 (EBV) or AJ871403.1 (VZV). Black bars in the outer edge represent masked repetitive regions and green bars genes. SNVs are marked with red (non-synonymous), green (synonymous) and blue (non-coding region) dots. Indels are marked with straight line. Details of the samples are shown in the corresponding tables.

The EBV sequenced from skin of the individual with MCL (breadth coverage 97%, depth coverage 47×) exhibited higher PS and H than any other herpesvirus in any other sample and as high as the average diversity of B19V (Figure 1B, C, Figure 5A, Supplementary Figure S1B, Supplementary Text S4, Supplementary Dataset S2). From the 37 MV mutations found within coding regions, the majority (59%) occurred in lytic phase genes. The non-synonymous to synonymous mutation ratio was 1.7.

Similarly to B19V, iSNVs in this patient′s EBV exhibited more C to T and G to A mutations than their counterparts, although this was not statistically significant (*P*= 0.10 and *P*= 0.42, respectively, two-side binomial test) (Supplementary Figure S3D).

The complete VZV genome reconstructed (breadth coverage 98%, depth coverage 32×) from the same skin sample contained 7 MVs, all of which were found within CDSs (Figure 5B and Table 1; Supplementary Dataset S2).

**Figure 6.**
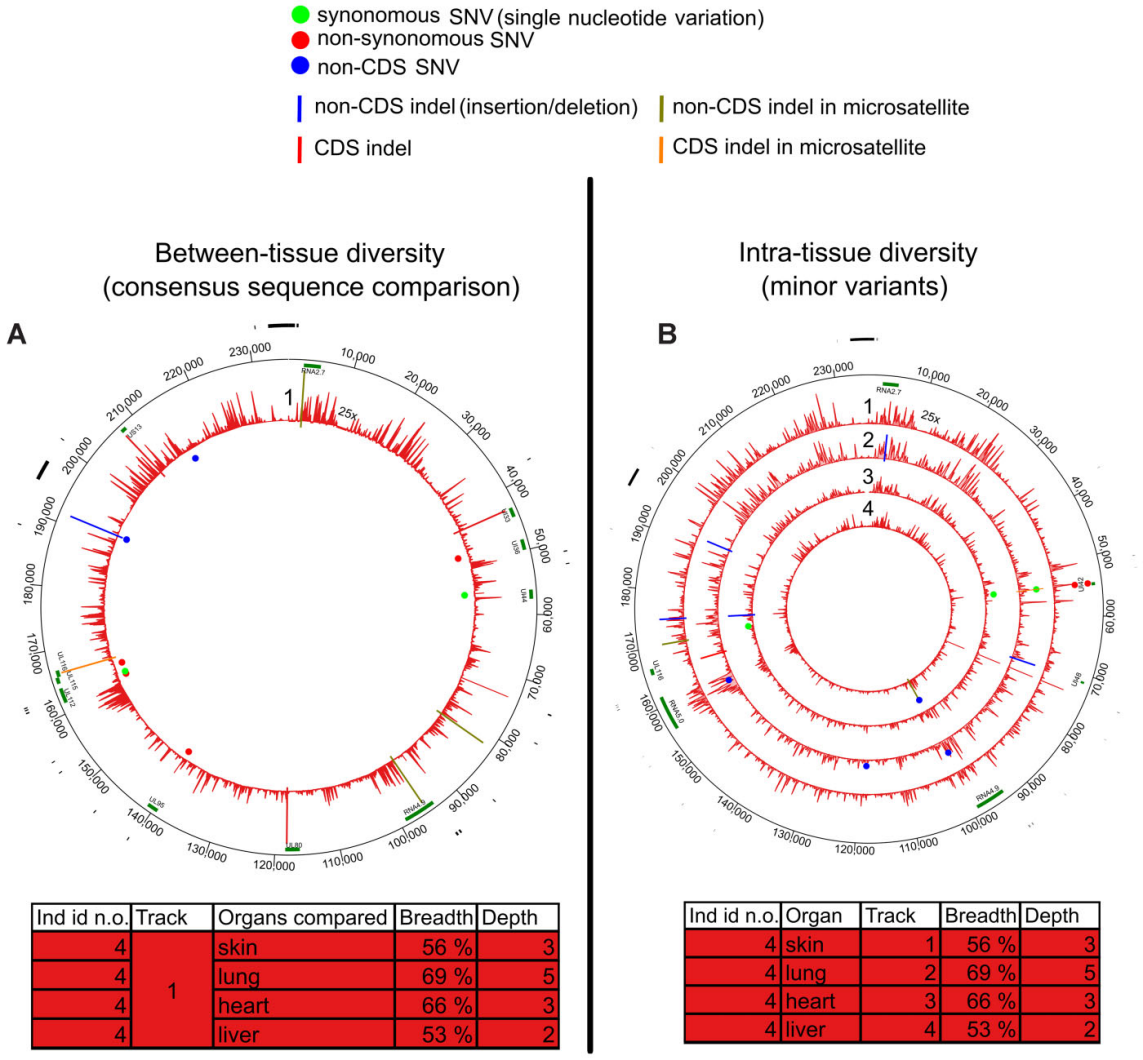
Intra-host diversity of HCMV in an individual with metastatic pulmonary carcinoma. (**A**) Comparison of viral consensus sequences between different organs of an individual. Circos plot track represents diversity in one individual, and the area illustrates the average depth coverage of the virus genomes (scale from 0 to 25×). (**B**) MV positions in each HCMV genome. Each track (numbered from 1 to 4) represents one sample, and the areas within tracks illustrate the depth coverage of the viral genome (scale from 0 to 25×). Both circos figures are plotted against the reference genome KJ361955.1. The black bars in the outer edge represent repeat regions masked from the analysis and the green bars genes. SNVs are marked with red (non-synonymous), green (synonymous) and blue (non-coding region) dots and indels with vertical lines. Details of the samples are shown in both respective tables.

From the other subject with terminal PC, we reconstructed genomes of HCMV from heart, liver, lung and skin (average breadth 61% and depth 3×) and of HSV-1 in blood, colon and lung (average breadth 67% and depth 5×). Both HCMV and HSV-1 exhibited higher intra-host diversity at the consensus-level and within samples than HHV-6B and -7 (Figures 1 and 6; Supplementary Figures S1 and S6, and Supplementary Text S5). The average MV frequencies were 35% for HCMV and 34% for HSV-1 (Supplementary Figure S2G and H). It is likely that their true diversity would be even higher with increased sequencing depth.

However, higher diversity was not reflected in all the virome constituents of these individuals: the JCPyV genomes reconstructed from the blood, colon, kidney, liver and lung (average breadth and depth coverage 96% and 39×) of the individual with PC exhibited no intra-host diversity between or within tissues. B19V was also reconstructed from six tissues of the individual with MCL (gt2; mean coverage 94%, 39×) and eight tissues of the one with PC (gt1; mean coverage 96%, 290×), with no detectable intra-host diversity.

### Distinction between co-infecting strains and minor variants of human papillomavirus

Although MVs can be identified robustly with short-read sequencing, discerning whether distant polymorphisms belong to a single or multiple haplotypes remains challenging, especially if they are partitioned by areas without polymorphisms (22). Thus, discriminating between standing variation and mixed infections, particularly among closely related strains, can be difficult (15,18,20).

We identified 22 MVs in the genome of a HPV type 23 (99.93% pairwise identity to type 23, reference KY652675.1) obtained from a hair sample. Upon closer inspection, we observed that all alternative nucleotides were present in close proximity to each other within the same sequence reads (Supplementary Figure S7A and B). By sequence alignment using BLAST, these alternative reads aligned more closely to HPV type 22 (100% identity to type 22, reference U31780.1), supporting a co-infection by both HPV types 23 and 22, rather than the presence of MVs of type 23 alone. Co-existence of these two HPV types was also supported by denovo contigs (Supplementary Figure S7C). We further realigned the sequencing reads against the HPV type 22 reference and confirmed by blasting that the reads indeed matched HPV type 22. Of note, neither *de novo* contigs nor the aligned reads provided any evidence of hybrid sequences, which would be indicative of recombination between these two HPV types, as opposed to co-infection (55).

### Viral integrations determined through virus-host split reads

Viral integration into the host genome can have diverse effects, ranging from promoting carcinogenesis by activating oncogenes or disrupting tumor suppressor genes, to causing genomic instability through insertional mutagenesis and chromosomal rearrangements. Additionally, viral insertions can alter gene expression, either by enhancing or silencing specific genes, and contribute to viral latency. Thus far, however, most studies on viral integrations have focused on malignancies, whereby the occurrence and relevance of such integrations in normal host tissues remains largely unknown.

We used a repeat-aware integration caller, SurVirus, to explore virus-host junctions (38). The detection of integration junctions is based on (i) read pairs in which the forward and reverse reads align to separate organisms (in this case one to a viral and the other to a human reference; supporting reads); (ii) hybrid reads of viral and human sequences (split reads) that contain the junction of the integration (Supplementary Figure S8).

As a positive control for integration screening from HTS data, we sequenced EBV from RAJI cell line (average breadth 99% and depth 124×) in which EBV integration has been characterized by molecular methods to be in 6q15 BACH2 gene (56). Concordantly, SurVirus identified a junction site between EBV BGRF1/BDRF1 and an intron of BACH2 in 6q15, supported by six reads. As a negative control, we mixed DNA of an EBV whole virus preparation (WHO standard, B95-8 strain) and a skin sample known to be EBV negative by both NGS and qPCR. We then sequenced EBV from this mixture (100%, 86×) from which SurVirus did not report any integrations.

**Figure 7.**
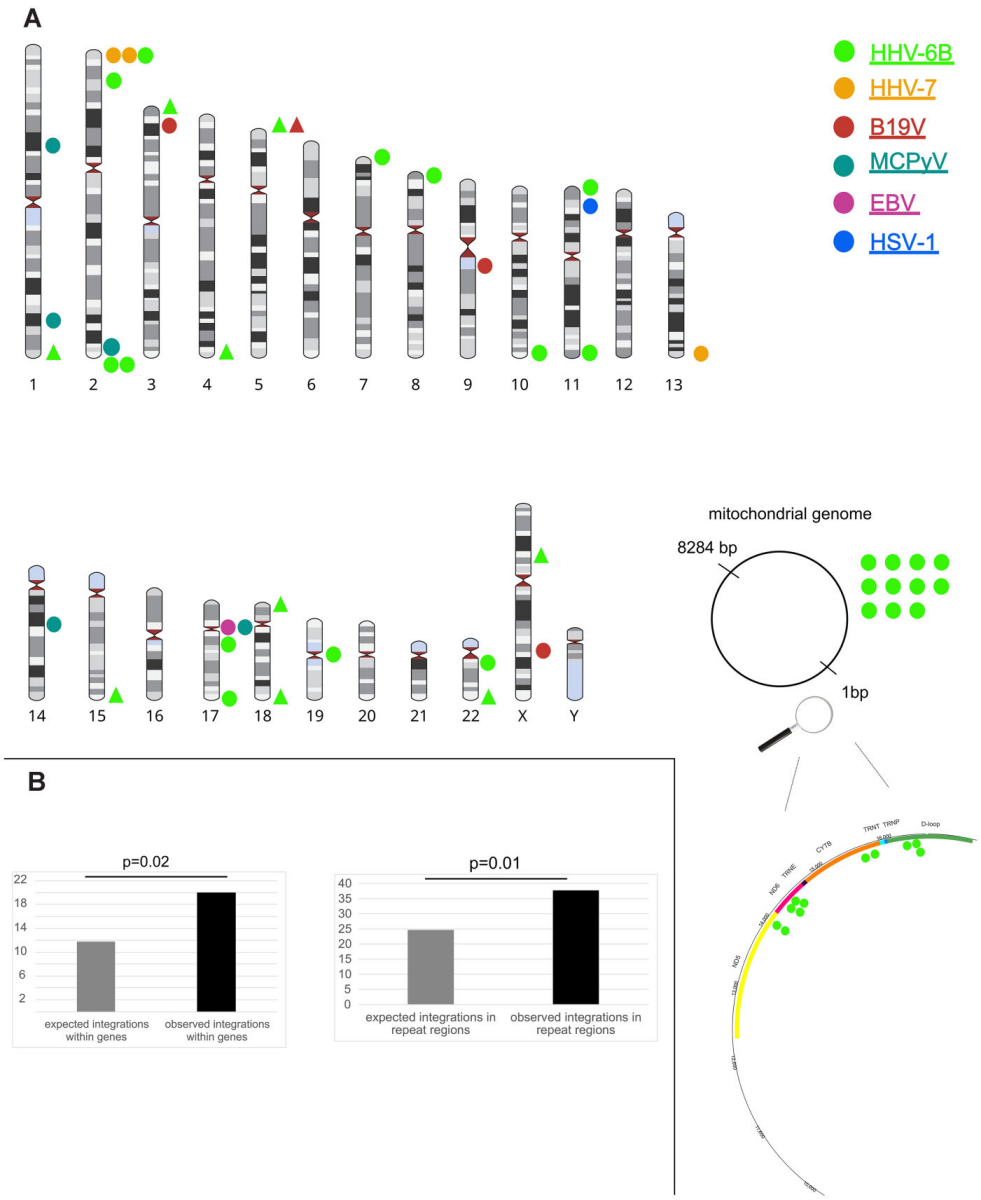
Karyogram illustrating all virus integrations reported by SurVirus. (**A**) On the top are all the human chromosomes accompanied by colored circles representing different virus species and their respective junctions. Triangles indicate integrations aligning equally to multiple chromosomes. At the bottom right is a mitochondrial genome with corresponding locations of HHV-6B junctions. (**B**) Comparison of expected (gray) and observed (black) number of integrations within genes (left) and in repeat regions (right). The *P*-value was calculated with chi-square test. Expected numbers were calculated assuming uniform random distribution of integrations based on the total length of genes and repeat regions in the human genome. B19V, human parvovirus B19; EBV, Epstein-Barr virus; HHV-6B, human herpesvirus 6B; HHV-7, human herpesvirus 7; HSV-1, herpes simplex virus 1; MCPyV, Merkel cell polyomavirus.

In our cohort, SurVirus reported a total of 47 integrations for 6 viruses (HHV-6B, HHV-7, B19V, MCPyV, EBV and HSV-1) from 127 genomes, backed by, on average, eight supporting reads and four split reads per junction (Figure 7A and Table 2; Supplementary Figure S8, Supplementary Dataset S3). SurVirus detected no integrations in any of ten JCPyV, four HCMV, VZV, HPyV6, HPyV7 or HPV type 23 genomes.

**Table 2.** Integration junctions identified with SurVirus

Id no	Organ	Virus	Host ref.	Host position	^a^Location	^b^ Location after BLAT	Host gene	Telomere	Repeat type	Virus ref	Virus position	Virus gene	c	d	Micro homology
4	Colon	B19V	hg38	-10601	5p15.33	5p15.33	NA	Yes	PTR	KM393164.1	4441	VP	6	3	A
4	Liver	B19V	T2T	90029475	xq21.31	xq21.31	NA		SINE	KM393164.1	2501	NS1	8	5	TTCC
30	Kidney	B19V	T2T	49368419	9q12	9q12	NA		hsat_3_sat	KM393165.1	4921	VP	8	3	TTC
30	Skin	B19V	hg38	-7075427	3p26.1	3p26.1	GRM7		SR (GT)n	KM393165.1	-381		6	2	
7	Hair	MCPyV	T2T	32788058	14q21.1	14q21.1	NA		LINE	HM011544.1	-748	VP2	8	3	
7	Hair	MCPyV	T2T	232182497	2q37.1	2q37.1	NA		SINE	HM011544.1	-766	VP2	6	7	
7	Hair	MCPyV	T2T	17947062	17p11.2	17p11.2	DRC3 ATPAF2		SR (TGTG)n	HM011544.1	3131	LT	8	8	
7	Hair	MCPyV	T2T	62229596	1p31.3	1p31.3	NA		SINE	HM011544.1	884	VP2	6	4	
7	Hair	MCPyV	T2T	-210706414	1q32.3	1q32.3	NA		Retroposon	HM011544.1	-2621	LT	6	4	
4	Colon	HSV-1	T2T	-9054629	11p15	11p15	TMEM9B-AS1		SINE	MH999850.1	-80092	UL36	8	4	
10	Skin	EBV	T2T	16433700	17p11.2	17p11.2	NA		SR (CA)n	MG298924.1	167136	LMP-1	6	6	CCTCT
11	Kidney	HHV-6B	hg38	80263076	18q23	multiple chr	NA	Yes	PTR	MH698401.1	8654	DRL	26	0	
11	Kidney	HHV-6B	hg38	-16164	mtDNA	mtDNA	d loop		SR (CAACTAT)n	MH698401.1	8654	DRL	14	12	AACCCAA
11	Kidney	HHV-6B	hg38	-248946276	1q44	chr1/2/3/4	NA	Yes	PTR	MH698401.1	-153340	DRR	16	1	
11	Kidney	HHV-6B	hg38	-240613841	2q37.3	2q37.3	CAPN10 GRP35	Yes	ITR	MH698401.1	8657	DRL	6	2	
11	Kidney	HHV-6B	hg38	-14516	mtDNA	mtDNA	NADH 6			MH698401.1	8653	DRL	10	8	AACCCA
11	Kidney	HHV-6B	hg38	14150	mtDNA	mtDNA	NADH 5			MH698401.1	-8190	DRL	6	5	TAACC
11	Kidney	HHV-6B	hg38	14427	mtDNA	mtDNA	NADH 6			MH698401.1	-161501	DRR	6	5	
11	Kidney	HHV-6B	hg38	-41800661	Xp11.4	Xp11.4 8q11.22/10q21.1	CASK/PXDNL		LINE	MH698401.1	161962	DRR	6	5	ACCC
11	Kidney	HHV-6B	hg38	15754	mtDNA	mtDNA	CYTB			MH698401.1	-8188	DRL	6	5	
11	Kidney	HHV-6B	hg38	-14130	mtDNA	mtDNA	NADH 5			MH698401.1	56766	U36	2	2	CTACTAC
11	Liver	HHV-6B	hg38	-10246	18p11.23	2p25.3	NA	Yes	PTR	MH698401.1	8653	DRL	6	4	
11	Lung	HHV-6B	hg38	-10813	5p15.33	multiple chr	NA	Yes	PTR	MH698401.1	8654	DRL	12	2	
6	Liver	HHV-6B	hg38	-11401	5p15.33	18p11.32 Yp11.2	NA	Yes	PTR	MH698401.1	8654	DRL	14	1	
7	Liver	HHV-6B	T2T	-63246846	11q12.3	19q12	AC005580.1 (lncRNA)		SR (CCCTT)n	MH698401.1	35596	U23	8	6	
7	Liver	HHV-6B	T2T	8032900	2p25.1	2p25.1	LINC00298		SR (A)n	MH698401.1	-18822	U10	6	3	
7	Liver	HHV-6B	hg38	-80262921	18q23	11q25 7p22.3 15p13	NA	Yes	PTR	MH698401.1	0	DRL	12	1	
30	Kidney	HHV-6B	hg38	-101981039	15q26.3	multiple chr	NA	Yes	PTR	MH698401.1	0	DRL	10	15	
30	Kidney	HHV-6B	hg38	14417	mtDNA	mtDNA	NADH 6			MH698401.1	-8191	DRL	6	4	
30	Kidney	HHV-6B	hg38	133765683	10q26.3	10q26.3	NA	Yes	SR (GTTAGG)n	MH698401.1	8653	DRL	6	4	
30	Kidney	HHV-6B	hg38	-16164	mtDNA	mtDNA	d loop		SR (CAACTAT)n	MH698401.1	8654	DRL	8	6	
30	Kidney	HHV-6B	hg38	133265066	12q24.3	8p23.3	NA	Yes	PTR	MH698401.1	8344	DRL	6	1	
30	Kidney	HHV-6B	hg38	-14384	mtDNA	mtDNA	NADH 6			MH698401.1	120	DRL	4	4	
30	Kidney	HHV-6B	hg38	15842	mtDNA	mtDNA	CYTB			MH698401.1	-153375	DRR	2	1	
27	Liver	HHV-6B	hg38	-78438426	17q25.3	17q25.3	DNAH17		SINE	MH698401.1	-155913	DRR	22	11	
27	Liver	HHV-6B	hg38	1756337	7p22.3	7p22.3	NA	Yes	PTR	MH698401.1	-161498	DRR	6	3	
27	Liver	HHV-6B	hg38	-50807904	22q13.33	multiple chr	NA	Yes	PTR	MH698401.1	0	DRL	8	0	
27	Kidney	HHV-6B	hg38	-240613747	2q37.3	2q37.3	CAPN10/GPR35	Yes	ITR	MH698401.1	300	DRL	6	4	
27	Kidney	HHV-6B	hg38	-16164	mtDNA	mtDNA	d loop		SR (CAACTAT)n	MH698401.1	8654	DRL	6	5	
16	Colon	HHV-6B	T2T	-31736399	17q11.2	17q11.2	CRLF3			MH698401.1	117991	U77	8	3	
19	Kidney	HHV-6B	T2T	-1077	3p26.3	3p26.3/3q29	NA	Yes	PTR	MH698401.1	-153340	DRR	6	5	
19	Lung	HHV-6B	T2T	202738	11p15.5	11p15.5	BET1L	Yes	PTR	MH698401.1	-8191	DRL	6	2	
19	Colon	HHV-6B	hg38	-190122941	4q35.2	multiple chr	NA	Yes	PTR	MH698401.1	-153340	DRR	6	3	
19	Colon	HHV-6B	hg38	-375172	22q11.21	22q11.21	NA		ct_22_98-sat	MH698401.1	132	DRL	6	2	
11	Colon	HHV-7	hg38	145076005	8q24.3	13q34	NA	Yes	PTR	AF037218.1	297	DRL	6	6	
11	Colon	HHV-7	hg38	-11582	5p15.33	2p25.3	NA	Yes	PTR	AF037218.1	848	DRL	6	1	
4	Liver	HHV-7	hg38	-11584	5p15.33	2p25.3	NA	Yes	PTR	AF037218.1	143894	DRR	6	3	

^a^Location reported by SurVirus.

^b^Location determined after BLAT search of junction adjacent host sequence.

^c^ and ^d^ indicate number of supporting reads and split reads, respectively. B19V, human parvovirus B19; DRR/L, direct repeat right/left; EBV, Epstein-Barr virus; HHV-6B, human herpesvirus 6B; HHV-7, human herpesvirus 7; HSV-1, herpes simplex virus 1; ITR, imperfect telomeric repeat; LINE/SINE, long/short interspersed nuclear element; MCPyV, Merkel cell polyomavirus; mtDNA, mitochondrial DNA; PTR, perfect telomeric repeat; sat, centromeric satellite; SR, simple repeat.

We discovered integrations in most human chromosomes (except 12, 16, 20, 21 and Y) as well as in mitochondrial sequences. A statistically significant proportion (81%) of integrations occurred in repetitive regions of the human genome (χ2, *P*= 0.01), (Figure 7B). The proportion of viral integrations occurring within genes (20/47, 43%) was also higher than expected by chance (χ2, *P*= 0.02) (Figure 7B). Integrations in chromosomal genes were found within introns.

### Multiple viral integrations seen in individuals with malignancy

Many DNA viruses, including EBV, have been shown to integrate into cancer cells, even though this is not part of the normal virus life cycle. SurVirus reported an integration event of an EBV genome, reconstructed from a skin sample of the individual with recently relapsed mantle cell lymphoma. This junction involved the EBV LMP-1 gene and chromosome 17 (17p11.2) (Figure 7A and Table 2). The fusion occurred through a shared ‘CCTCT’ sequence, likely via microhomology-mediated end joining (MMEJ) (Supplementary Figure S9) (45).

In the individual with metastatic pulmonary carcinoma, SurVirus reported integration of HSV-1 UL36 gene and TMEM9B.AS1 long non-coding RNA (lncRNA) in chromosome 11, in a colon sample. HSV-1 integrations have been similarly solely reported in cancer cell lines under unusual circumstances (57,58).

### Scarcity of human parvovirus B19 and host junctions undermines integration as primary mechanism for lifelong persistence

The mechanism of B19V DNA persistence in human tissues has remained enigmatic. Integration has been speculated as a potential strategy since other parvoviruses such as adeno-associated virus (AAV) have this capability. Integration of B19V has been previously reported *in vitro* in its primary infection target, erythroid progenitor cells (EPCs), with the NS1 protein facilitating integration via nicking of host and viral DNA (59). However, during *in vivo* persistence, B19V DNA resides in cells other than EPCs, such as endothelial cells, monocytes and B cells (60).

In the individual with pulmonary carcinoma, SurVirus identified an integration junction of B19V in the colon and liver. Additionally, only one other individual (without reported cancer) exhibited B19V integrations in the kidney and skin. Notably, the virus junction in the skin sample occurred in close proximity (16 bp) to a sequence in which the viral NS1 nicks DNA (terminal resolution site), supporting the possible role of this protein in integration facilitation. However, based on the low prevalence of samples with integrations (4 out of 60), our data indicate that this is not the predominant mechanism for lifelong tissue persistence of B19V (59,61).

### Merkel cell polyomavirus integration and LT gene truncations present in cancer free individuals

MCPyV is an oncogenic virus causing 80% of Merkel cell carcinomas (MCCs). In all those cases, MCPyV occurs clonally integrated, with its large T antigen (LT) truncated. This truncation leads to the loss of LT motifs necessary for viral replication, while retaining the RB-binding motif driving oncogenicity. Both events are required for malignant transformation (62). In our cohort, among the eight samples from which MCPyV genomes were reconstructed, SurVirus identified five integration breakpoints in one sample of plucked hairs (Figure 7A and Table 2) from an individual without malignancies.

We investigated MCPyV LT truncations using the structural variant prediction method DELLY (46). From the same sample in which MCPyV integrations were detected, DELLY reported a minor subset of reads with a deletion between positions 889 (VP2) and 4220 (LT), and between 1619 (VP1) and 5123 (LT/ST), (Supplementary Figure S10A). The former deletion preserved the RB binding motif of LT analogously to the truncations seen in MCC.

In plucked hairs of another individual, DELLY identified, in a minor subset of reads, a substantial deletion between positions 1430 and 4452, spanning from VP1 to LT. However, this truncation also resulted in loss of the RB-binding motif. This deletion was supported by both split reads and paired-end analysis, as well as by *de novo* assembly (Supplementary Figure S10B and C).

We also used DELLY to screen for structural variants in other polyomaviruses (four JCPyV and one HPyV6 genomes) and in 25 B19V genomes, albeit observed no similar deletions.

### Human herpesvirus 6B prevails within integration landscape of normal tissue virome

HHV-6B is the only DNA virus in this cohort known to establish latency through integration; this occurs between viral telomeric repeats and host subtelomeric regions. However, with the closely related HHV-6A, also non-telomeric integrations have been reported (63). Expectedly, most integrations identified by SurVirus were related to HHV-6B: in total 33 integrations across 7 individuals and 12 samples (Figure 7A).

Five different types of junctions were identified: (i) virus unique (U) region to non-telomeres of the host (three junctions), (ii) virus telomere to non-telomeres of the host (five junctions), (iii) virus telomere to host telomeres/subtelomeres (14 junctions), (iv) virus telomere to mitochondrial DNA (mtDNA), (10 junctions) and (v) Virus U region to mtDNA (1 junction) (Table 2).

HHV-6B integrations in mitochondrial sequences occurred in three kidney samples. One of these junctions was between the unique region (U36 gene) of HHV-6B and the mtDNA NADH dehydrogenase subunit 5 (MT-ND5). The remaining junction sites consisted of split reads of HHV-6B telomeric repeats and MT-ND5 (1 junction), MT-ND6 (4 junctions), cytochrome B (2 junctions) or D-loop (3 junctions) of the mtDNA.

## Discussion

Following primary infection, many DNA viruses establish lifelong persistence. However, the temporal evolutionary dynamics across decades, as well as the within-host diversities remain poorly understood. We previously characterized the landscape of DNA viruses persisting in nine organs of multiple individuals, demonstrating a high degree of conservation between different anatomical compartments of the body (3). Building upon these findings, in the present study we examined the viral sequences from the tissues of 13 individuals, to analyze in-depth the inter- and intra-organ diversities of persisting viral genomes. Moreover, we investigated *in silico* the occurrence of viral integrations within the human genome (38), as a potential mechanism underlying viral lifelong persistence within human cells.

This work is unique in several aspects: Unlike most research on the topic, which typically rely on clinical cohorts with specific conditions (8,12,14,15,21,64–68), our data reflect more closely the intra-host diversities of viruses persisting among the general population. Moreover, our analysis encompasses the eukaryotic DNA virome across multiple solid tissues of the human body, while the existing studies have focused on single species, typically during active replication, in restricted anatomical compartments (primarily bodily fluids).

Consequently, the present study provides an essential foundation on the genetic footprint of persistent viruses at a steady state, laying a valuable framework for the evaluation of shifts in diversity arising from selective pressure or pathological conditions. Accordingly, our findings provide novel insights into the intra-host evolution of viruses as well as their organ tropism, vital in addressing antiviral resistance (69,70), immune evasion (71) and pathogenicity (8–10,12,13,72).

Our analysis revealed minimal inter-organ variations within an individual, with consensus sequences in most cases featuring no mutations or a maximum of 18 per virus species. This suggests that long-term tissue persistence likely originates from a sole founding virus strain and that any tissue-specific selective pressures or potential adaptive changes across organs are marginal. Alternatively, the similarity of viral sequences between different tissue types may point to comparable cellular environments or equivalent host cells, such as resident lymphocytes homing to different organs (60,73).

The limited variability likely represents a gradual decline in intra-host diversity over decades. This is based on the assumption that most primary infections typically occur during childhood and that the average age of our cohort was 71 years. This phenomenon has been noted in previous studies, such as for B19V, for which a substantially lower mutation rate during persistence (as opposed to acute infection) has been reported (74). Similarly, for EBV, Weiss *et al.* (75) reported a decrease in intra-host genetic diversity overtime following infection, with convergence towards a common sequence.

Remarkably, in some viral species we observed clear asymmetry on the mutational spectra towards specific transitions, specifically from C to T and G to A. This was the case for B19V, particularly in the colon, the organ with the highest expression of APOBEC-3B cytidine deaminase among all the organs analyzed in this study (76). This is in line with previous investigations demonstrating that especially small DNA viruses, such as B19V, are targeted by APOBEC3 (77). Our findings support a potential role for this enzyme in shaping viral evolution and persistence, and in modulating long term host-virus interactions.

In addition to the consensus-level comparisons, we performed MV analyses to identify genomic positions shared by more than one nucleotide. MVs represent standing genetic variation within viral populations, providing a reservoir for rapid adaptation to changes in the environment, such as selective pressure by antiviral medication (15).

In the majority of viral genomes examined, MVs were either absent or present maximally at 10 per genome, indicating minimal genetic intra-host diversity. This finding further supports the establishment of persistence by a single rather than multiple strains (20). However, although our enrichment protocol has been optimized to capture highly divergent sequences, the bait design used in this study was primarily based on single references, whereby, different strains may be captured with varying efficiency. Therefore, genotyping of highly polymorphic regions may be valuable to further characterize the level of diversity in the tissues.

To the best of our knowledge, we describe, for the first time, MVs of parvovirus B19 (B19V), MCPyV and HHVs 6B and 7, alongside those previously reported for other herpesviruses (21,66–68,75,78–84). In the present study, the intra-organ diversities were minimal, in the order of 10^−6^ for HHV-6B and HHV-7, two magnitudes higher for B19V, and three for MCPyV. These findings align with a previously demonstrated inverse correlation between viral diversity and genome size (85). Apart from the highest number of MVs, MCPyV also exhibited a greater ratio of single nucleotide variations (SNVs) compared to insertions/deletions and equal diversity between coding and non-coding areas. In contrast, the other viruses typically had mutations enriched outside of genes. This increased diversity might reflect chronic replication and continuous shedding of MCPyV from hair and surrounding skin, (86) also supported by the extremely high copy numbers of viral genomes detected in the plucked hair samples of this cohort (range ∼10 000 to 10 000 000 copies per million cells) (3).

In the present study, based on 60 whole genomes, B19V variability was confined exclusively to genotype 2 (six individuals, nine tissues). In three individuals, we detected novel indels between and within organs in the inverted terminal repeats (ITRs) that are required for initiation of viral replication (87). Given that these indels may introduce instability into the complementary structures (self-folding), it is intriguing to speculate whether alterations in the ITRs could have played a role in the extinction of this genotype (60,88–90). The higher diversity of genotype 2 is also supported by a study of Bichicchi *et al.* on 24 highly-viremic sera (91).

Reactivations of latent viruses during a person's life could affect intra-host diversity, at least in the short term. In our cohort, two individuals likely had concomitant viral reactivations (in one of EBV and VZV and in the other of HSV-1 and HCMV (3)) suggested by extremely high quantities (up to 900 000 copies per million cells), and widespread distribution. Notably, all these viral genomes exhibited MVs, with as many as 51 detected in the EBV genome reconstructed from a skin sample of a patient with recently relapsed mantle cell lymphoma. While the lymphoma may have prompted selective pressure on EBV (11,72,92), the role that the increased viral diversity may have played on the transformation of this malignancy (72,93–95) deserves further examination. Indeed, while the greatest variability of EBV has earlier been observed in the latent genes (75), we found the majority of intragenic MVs in lytic phase genes, which have been linked to lymphomagenesis (72,96).

Worth noting is the fact that the high viral loads found in these two patients could have enhanced the detection of viral subpopulations. Hence, comparative studies of MVs from latent viruses in both health and disease should address the true biological impact of viral genetic diversity on the onset, progression and outcome of diseases.

In addition to the whole genome diversity analyses, HTS has enabled the screening of virus-host junctions (24,97). This approach has been primarily used in the context of malignancies (4) with focus on HPV (44,98,99), HBV (27–29,100), MCPyV (62,101), or EBV (102–104). Our present study, in line with prior findings, reveals several key insights: First, viruses can have integrations at multiple sites in a sample. Second, these integrations are distributed widely across all chromosomes, preferentially in introns of host genes and unstable genomic regions, such as low-complexity or repeat areas. Third, most of these junctions appear to be rare, as evidenced by the low number of junction-supporting reads relative to total viral reads. This suggests that insertions take place only in a minority of cells, in contrast to the clonal expansion observed in tumors (4,27–29,44,99–101,103). The rarity of specific junctions indicates that most integrations are stochastic, potentially resulting in a replication-incompetent dead-end for the virus or even in cell death (102).

Using stringent filters in the repetitive-aware integration caller SurVirus (38), we identified altogether 47 breakpoints for EBV, MCPyV, HHV-6B, B19V, HHV-7 and HSV-1. Previous literature on integration calling from tumor samples have reported as much as hundreds of breakpoints per sample, albeit in most cases a junction is supported by only few chimeric reads (27–29,98,102).

In the labile chromosomal area 17p11.2, we detected integrations of the well-known oncogene EBV-LMP1, as well as of the large tumor antigen gene of MCPyV, each in one individual. Integrations into this locus have been previously reported for EBV, HPV and HBV in malignant tissues (27,98,102). Aberrations at this site can lead to the formation of isochromosome 17q, implicated in the development and progression of multiple malignancies (105–107). Together these findings underscore an increased frequency of viral integrations among chromosomal fragile areas and repetitive regions (24,29,92,102,103,108–110). Whether these areas are more susceptible to viral insertions, or if viral integration induces genomic instability warrants further investigation (110).

Clonal integration and specific truncations of the MCPyV LT gene are prerequisites for malignant transformation in 80% of MCCs (62,101,111–113). Remarkably, we observed MCPyV LT truncations in hair specimens of two cancer-free individuals, while one of these subjects also exhibited five integration junctions. This suggests that both integrations and truncations can occur even in healthy subjects and may precede the development of MCC (114,115).

While integrations of HHV-6B typically occur in human telomeres via viral telomeric repeats (116), our analysis yielded unexpected evidence of insertions into mtDNA. Notwithstanding the challenge to distinguish between nuclear mitochondrial (NUMT) and authentic mtDNA with short read sequencing, similar junctions have been recently reported for HBV (30,100,117,118), and AAV (119). Most importantly, herpesviruses, such as HSV-1 (120), HHV-6A (121) and HHV-6B (122), can disrupt the mitochondrial network causing structural abnormalities and fragmentation, as well as induce the release of mtDNA into the cytosol (120). The resulting stress responses upregulate interferon type 1 and APOBEC3, which not only participate in antiviral response but also mediate mtDNA catabolism (120). Considering the potential implications of HHV-6B in exacerbating mitochondrial diseases, further investigations are warranted to explore the impact of the observed integrations on mitochondrial dynamics (122,123).

While the results presented are supported by both positive and negative controls, confirmation of the integration sites by PCR and Sanger sequencing was unfeasible due to limited sample availability. Nevertheless, previous literature has shown good agreement between similar *in silico* screenings and validation methods (27,29,38,97,98,103,108).

In conclusion, we analyzed the intra-host variability of the human tissue-resident virome. The viral sequences recovered from multiple organs within each individual were mostly conserved, indicating that a given virus can persist within distinct body compartments in the absence of tissue-specific mutations. Our research revealed the presence of MVs the extent of which greatly varied between samples, individuals, virus species and clinical contexts. Moreover, we explored the integration landscape, demonstrating that integrations of persistent viruses can also occur in the absence of malignancy.

Ultimately, our work showcases the value of thorough genetic analysis of viral whole genomes from tissues to comprehend the evolutionary forces shaping the virome. This blueprint is essential for evaluating deviations in viral diversity and genomic viral–host interactions emerging from or leading to disease.

## Supplementary Material

gkae871_Supplemental_Files

## Data Availability

The consensus sequences (*n* = 33) with the highest coverage (>90% breadth) from each individual are available at GenBank with accession numbers ON023008-ON023041. The raw sequencing reads for all samples can be accessed at the NCBI sequence read archive (SRA), accession PRJNA924035. The code of TRACESPipe is available on Zenodo (DOI: 10.5281/zenodo.7646369). The data underlying this article will be shared on reasonable request to the corresponding author.
