# Supplementary material for: Intra-host genomic diversity and integration landscape of human tissue-resident DNA virome
Source: Nucleic Acids Res. 2024 Oct 22;52(21):13073–93. doi: 10.1093/nar/gkae871 (PMC11602146; doi:10.1093/nar/gkae871)
Supplement: gkae871_Supplemental_Files [file gkae871_supplemental_files.zip › Supplementary_file-amendment-pdf.pdf]

Supplementary Figures

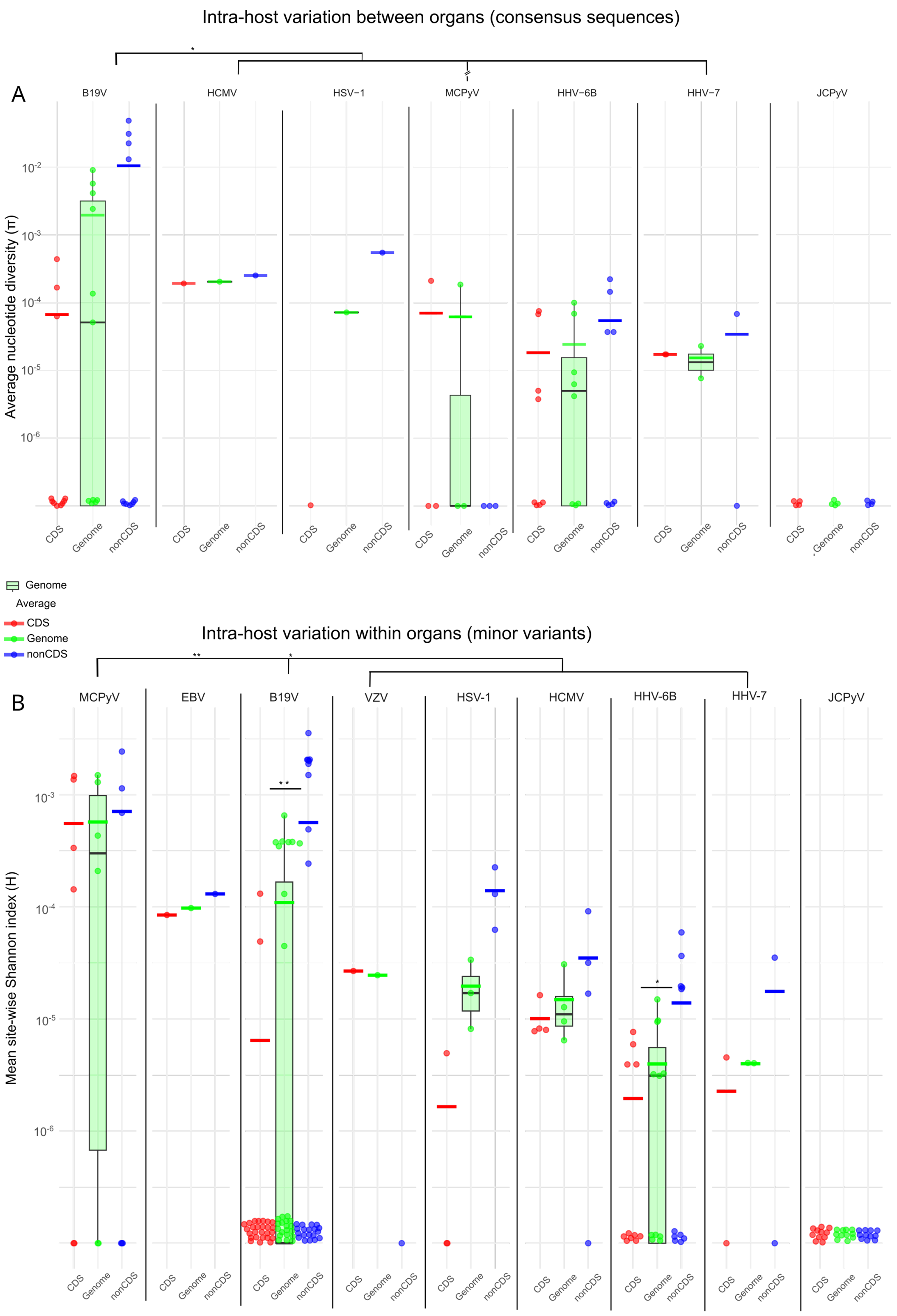

Supplementary Figure S1. Alternative metrics for intra-host genetic diversity

***A**) Average nucleotide diversity (π) when comparing viral genomes between organs within individual. Each datapoint represents nucleotide diversity in one individual.*

***B)** Mean site-wise Shannon index (H) calculated from the number and frequency of minor variants. Each datapoint represent one sample.*

*Different viruses are divided into separate panels, each containing a separate plot for CDS (red), whole genome (green) and non-CDS (blue). Horizontal lines illustrate the mean values. For the whole genome, a boxplot is shown. Datapoints at the bottom represent zero-diversity. CDS and non-CDS values within each virus species were compared with a permutation test of the means, with significant results shown in the figure. Whole genome diversity values were compared between each virus using a permutation test of the means, with Bonferroni correction for multiple comparisons. The brackets indicate a comparison of B19V or MCPyV to herpesviruses as a single group. * $p<0.05$, ** $p<0.01$.*

MCPyV=Merkel cell polyomavirus, EBV = Epstein-Barr virus, B19V = human parvovirus B19, HCMV = human cytomegalovirus, VZV = varicella-zoster virus, HSV-1 = herpes simplex virus 1, HHV-6B = human herpesvirus 6B, HHV-7 = human herpesvirus 7, JCPyV = JC polyomavirus

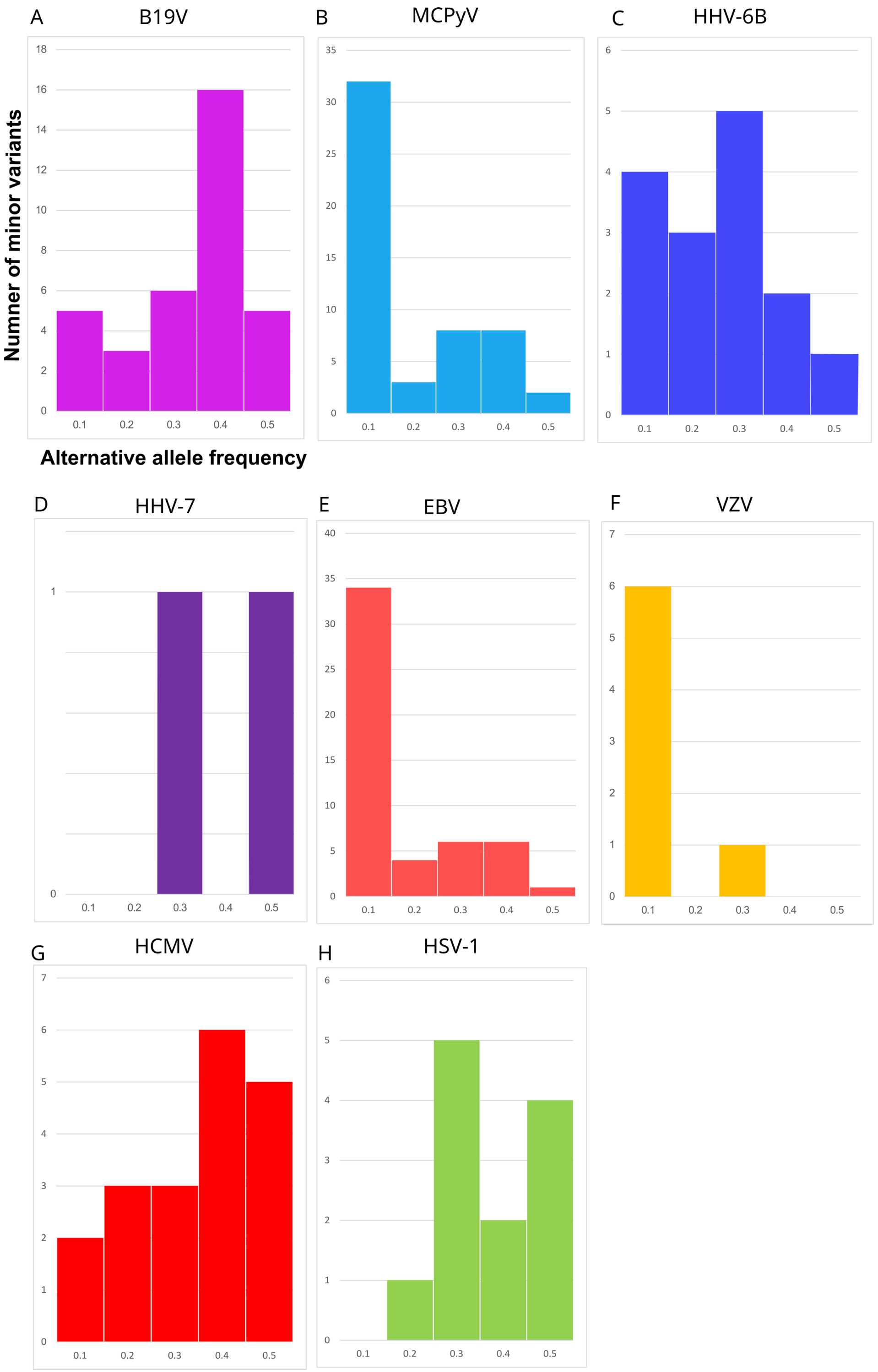

Supplementary Figure S2. Site frequency spectrum of minor variants. *The Figure shows the number of minor variants (y-axis) and their respective frequencies (x-axis) for all viruses. Each minor variant was assigned to frequency groups of 0.1-0.5 based on the rounded frequency of the variant. Minor variants with frequency of 0.03-0.09 were rounded up as 0.1.*

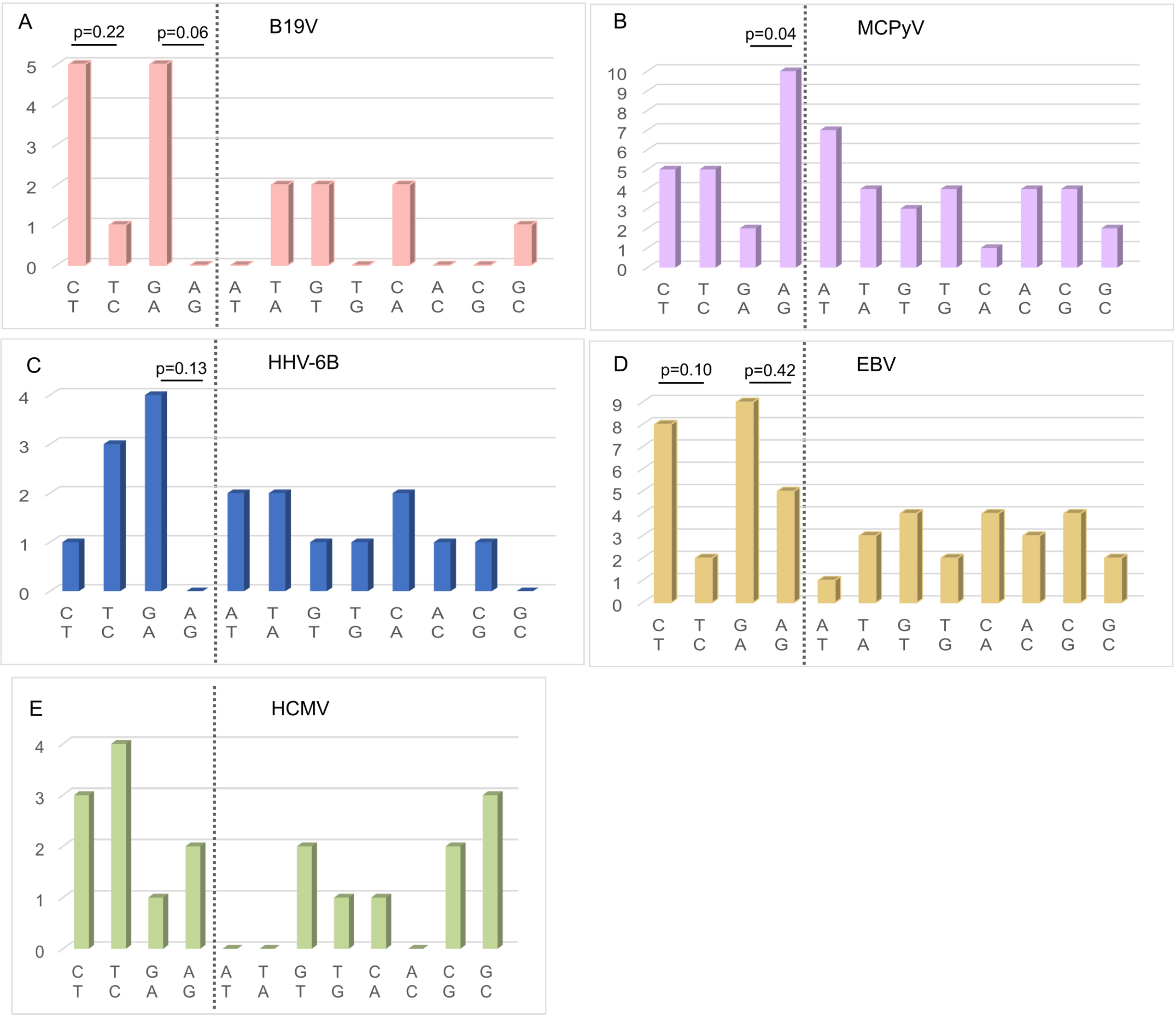

Supplementary Figure S3. *Within host virus transitions and transversions. Figure illustrates the distribution of cSNVs and iSNVs to different mutation types. On the x-axis are different substitutions divided into four transitions (left) and eight transversions (right), divided by a dashed line. The y-axis represents the number of both cSNVs and iSNVs for* ***A)*** *B19V,* ***B)*** *MCPyV,* ***C)*** *HHV-6B,* ***D)*** *EBV, or* ***E)*** *HCMV. For EBV data is only derived from iSNVs of a single sample. The number of C to T or G to A mutations were compared to their counterparts by two-sided binomial test. P-value showed above bars.*

A

5'ITR position 65: **TACGTCATTTCCTAT / G** ACGTCATTTCCTAT / GACGTCATTTCCT**G**T / GACGTCA**C**TTCC**G**GT

5'ITR position 275: **C**GGAA**G**TGACGTCA**C** / **AGGAAATGACGTCAT** / AGGAAATGACGTCAT / AGGAAATGACGT**AG**T

3'ITR position 5288: **ACGTCATTTCCTATG** / ACGTCATTTCCTATG / ACGTCATTTCCT**G**TG / ACGTCA**C**TTCC**GG**TG

3'ITR position 5498: **C**GGAA**G**TGACGTCA**C** / **AGGAAATGACGTCAT** / AGGAAATGACGTCAT / AGGAAATGACGT**AG**T

B

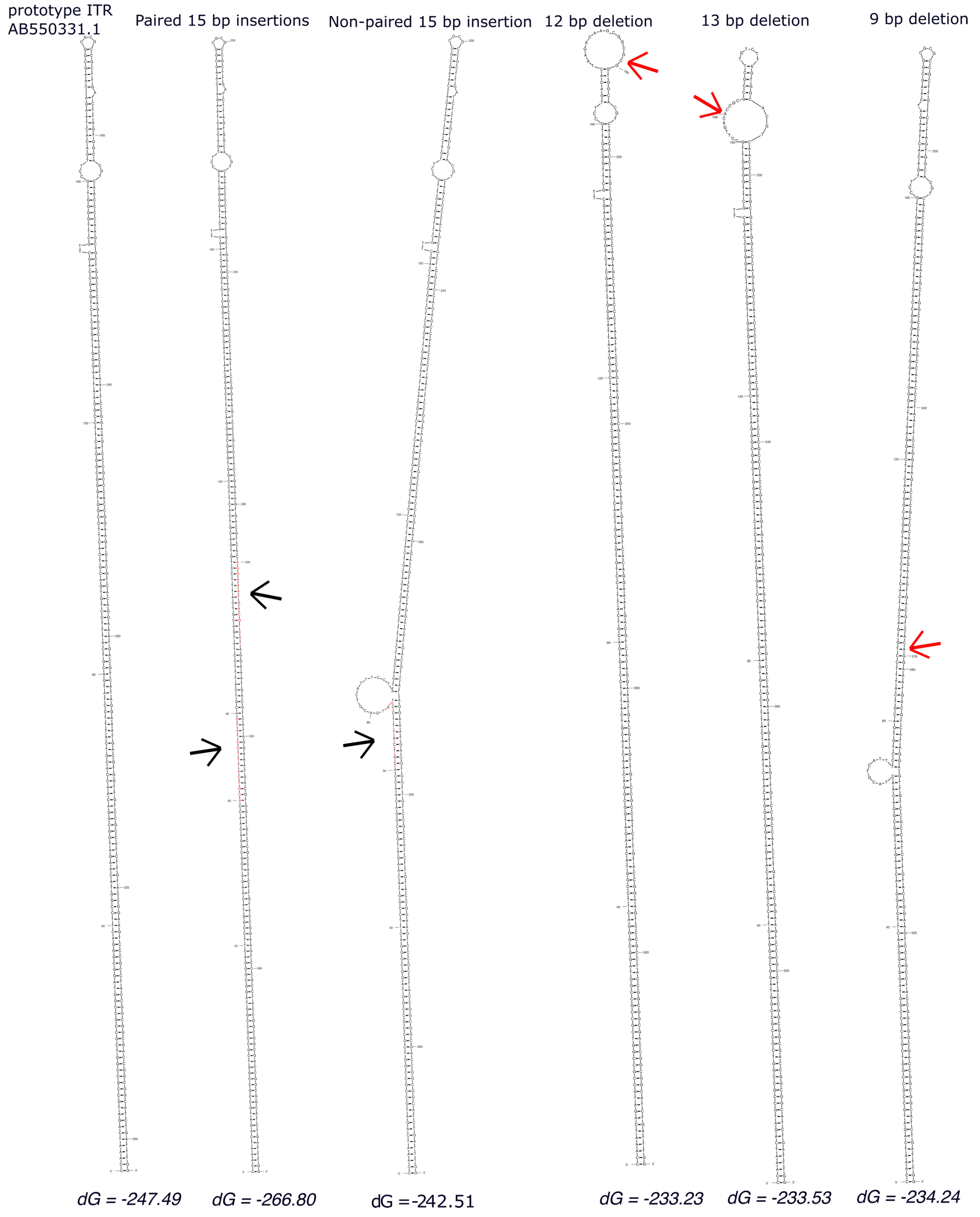

Supplementary Figure S4. ***Illustration of indels in B19V inverted terminal hairpins (ITRs). A)*** *Sequence of 15-nt insertions (in underlined red) within imperfect repeat sequence of ITR.* ***B)*** *5'end ITR self-folding prediction based on (from left to right) the 1) prototype sequence (AB550331.1) without indels, 2) sequence with 15-nt insertion as pairs, 3) non-paired 15-nt insertion 4) sequence with deletion of 12-nt or 5) 13-nt and 6) deletion of 9-nt. Insertion locations are marked with black arrows and deletions with red arrows. Below are free energy predictions (Gibb's free energy, negative values) with lower value (higher absolute value) indicating a more thermodynamically stable structure. Nucleic acid folding prediction made with Mfold web server.*

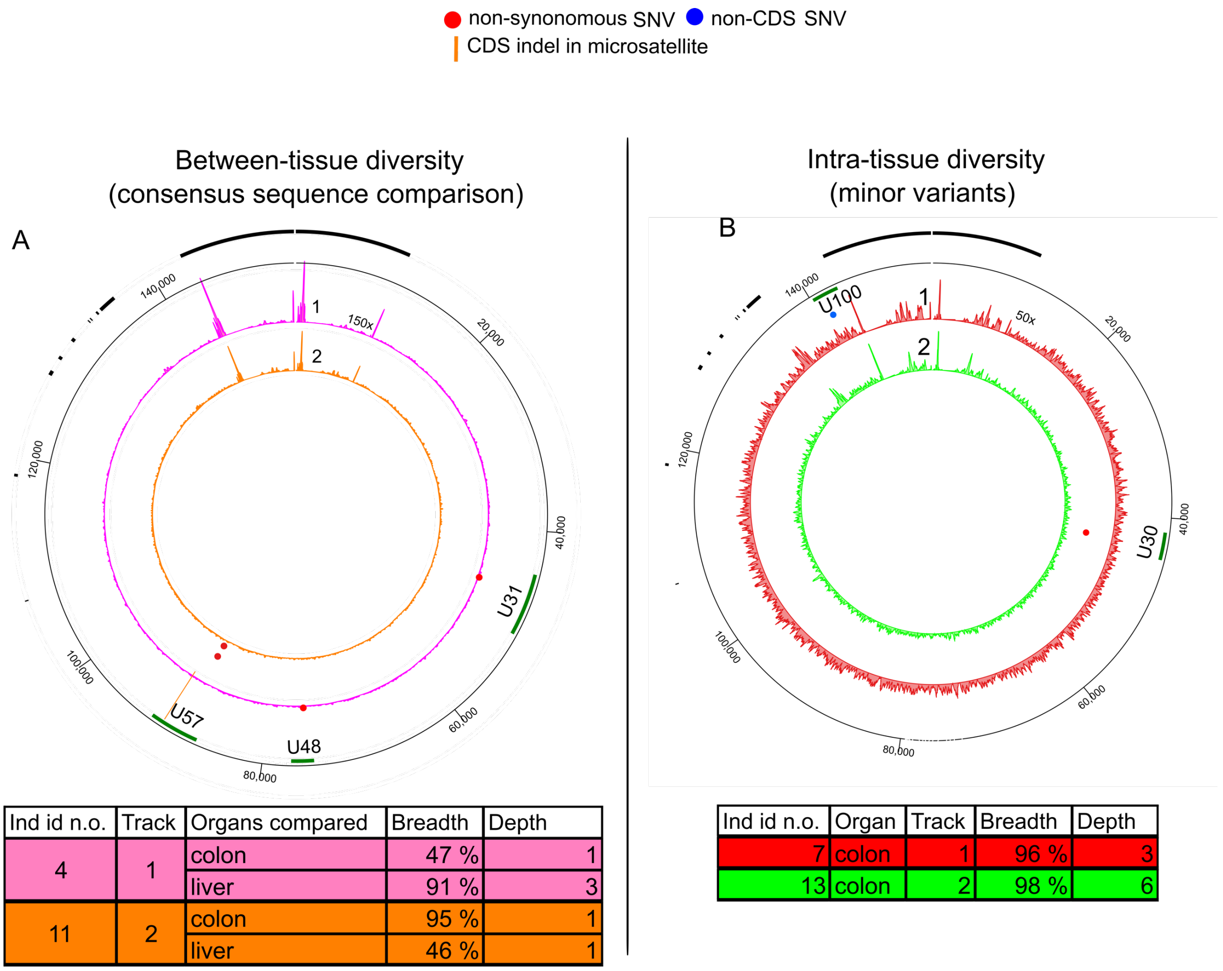

Ind id n.o.	Track	Organs compared	Breadth	Depth
4	1	colon	47 %	1
		liver	91 %	3
11	2	colon	95 %	1
		liver	46 %	1

Ind id n.o.	Organ	Track	Breadth	Depth
7	colon	1	96 %	3
13	colon	2	98 %	6

Supplementary Figure S5. Intra-host variation of HHV-7.

A) *Comparison of viral consensus sequences between different organs within an individual. Each circos plot track (numbered from 1 to 2) represents diversity in one individual, and the area illustrates an average coverage depth of the virus genomes (scale from 0 to 150X).*

B) *Minor variant positions in each HHV-7 genome. Each track (numbered from 1 to 2) represents one sample. Samples from which minor variants were identified are different than samples in panel A. The areas within tracks illustrate the coverage depth of the viral genome (scale from 0 to 50X).*

Both circos figures are plotted against reference genome AF037218.1. The black bars in the outer edge represent repeat regions masked from the analysis and the green bars genes. Single nucleotide variations (SNVs) are marked with red (non-synonymous), and blue (non-coding region) dots and indels with vertical lines. Details of the samples are shown in both respective tables below.

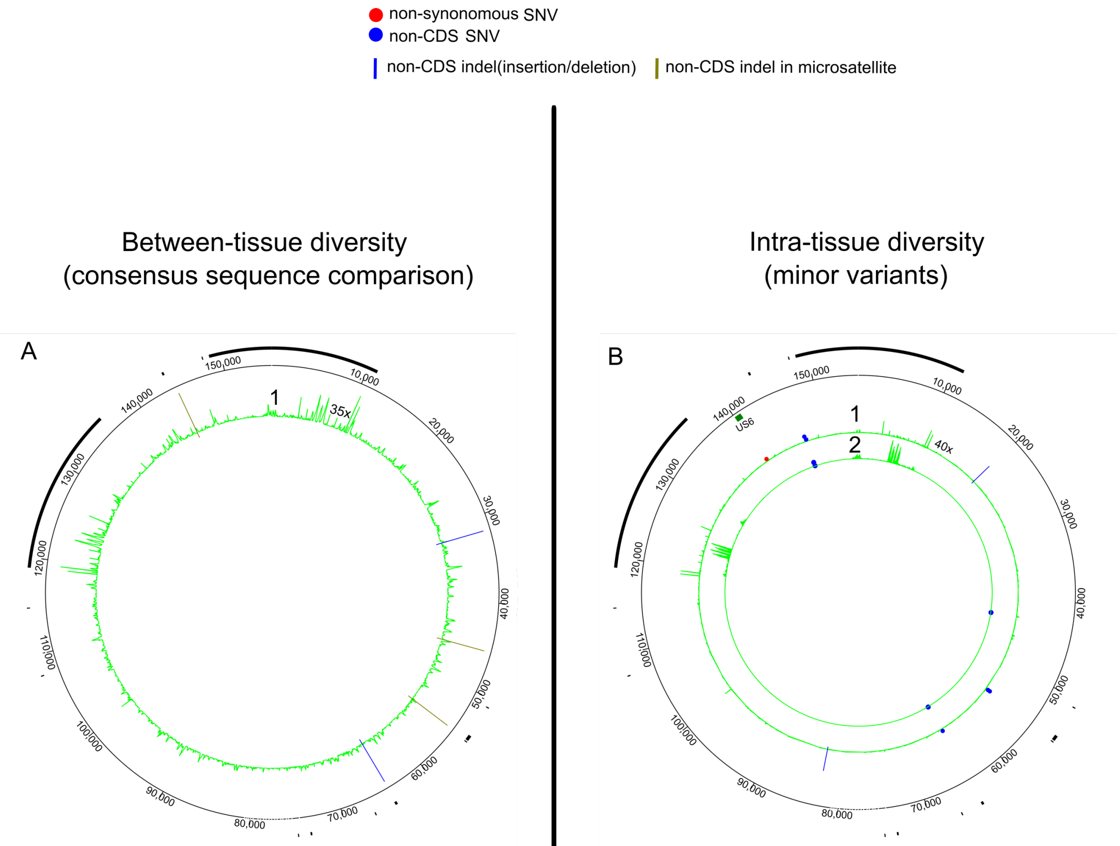

Ind id n.o.	Track	Organs compared	Breadth	Depth
4	1	blood	91 %	5
		colon	57 %	4
		lung	52 %	7

Ind id n.o.	Track	Organs compared	Breadth	Depth
4	1	lung	52 %	7
	2	blood	91 %	5

Supplementary Figure S6. Intra-host variation of HSV-1.

A) *Comparison of viral consensus sequences between different organs within an individual. Circos plot track represents diversity in one individual, and the area illustrates an average coverage depth of the virus genomes (scale from 0 to 35X).*

B) *Minor variant positions in each HSV-1 genome. Each track (numbered from 1 to 2) represents one sample. The areas within tracks illustrate the coverage depth of the viral genome (scale from 0 to 40X).*

Both circos figures are plotted against reference genome MH999850.1. The black bars in the outer edge represent repeat regions masked from the analysis and the green bars genes. Single nucleotide variations (SNVs) are marked with red (non-synonymous), and blue (non-coding region) dots and indels with vertical lines. Details of the samples are shown in both respective tables below.

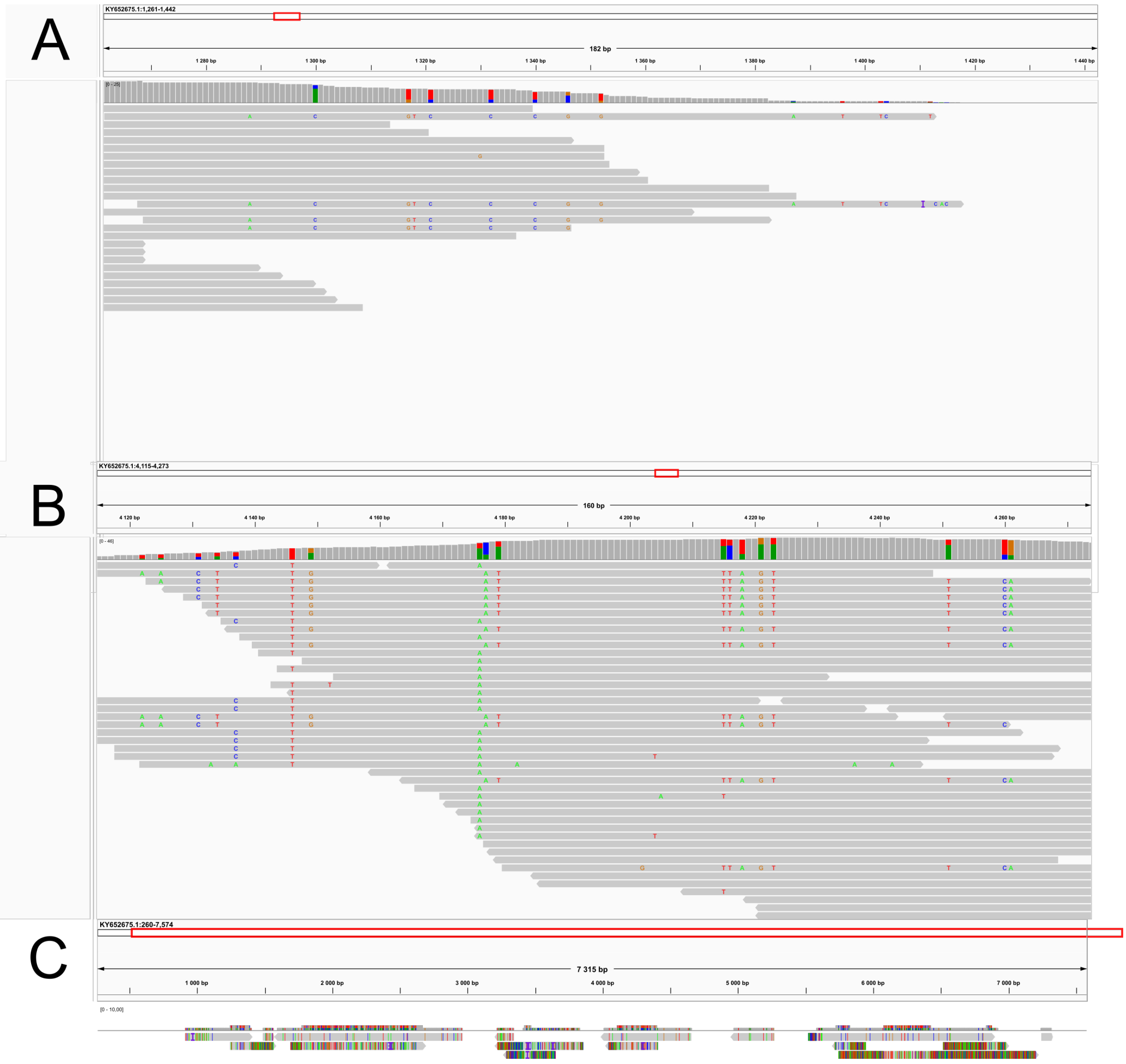

Supplementary Figure S7. *Reads aligned against HPV type 23 reference KY652675.1. The figure shows minor variants detected in A) region 1261-1442 and B) 4115-4273. All proximal alternative nucleotides existed within the same reads, indicating they represent a single haplotype. Blasting of these reads showed that they matched to reference HPV type 22 (U31780.1). C) Illustrates denovo scaffolds that match either to type 23 (close match to reference) or type 22 (many differences to the reference).*

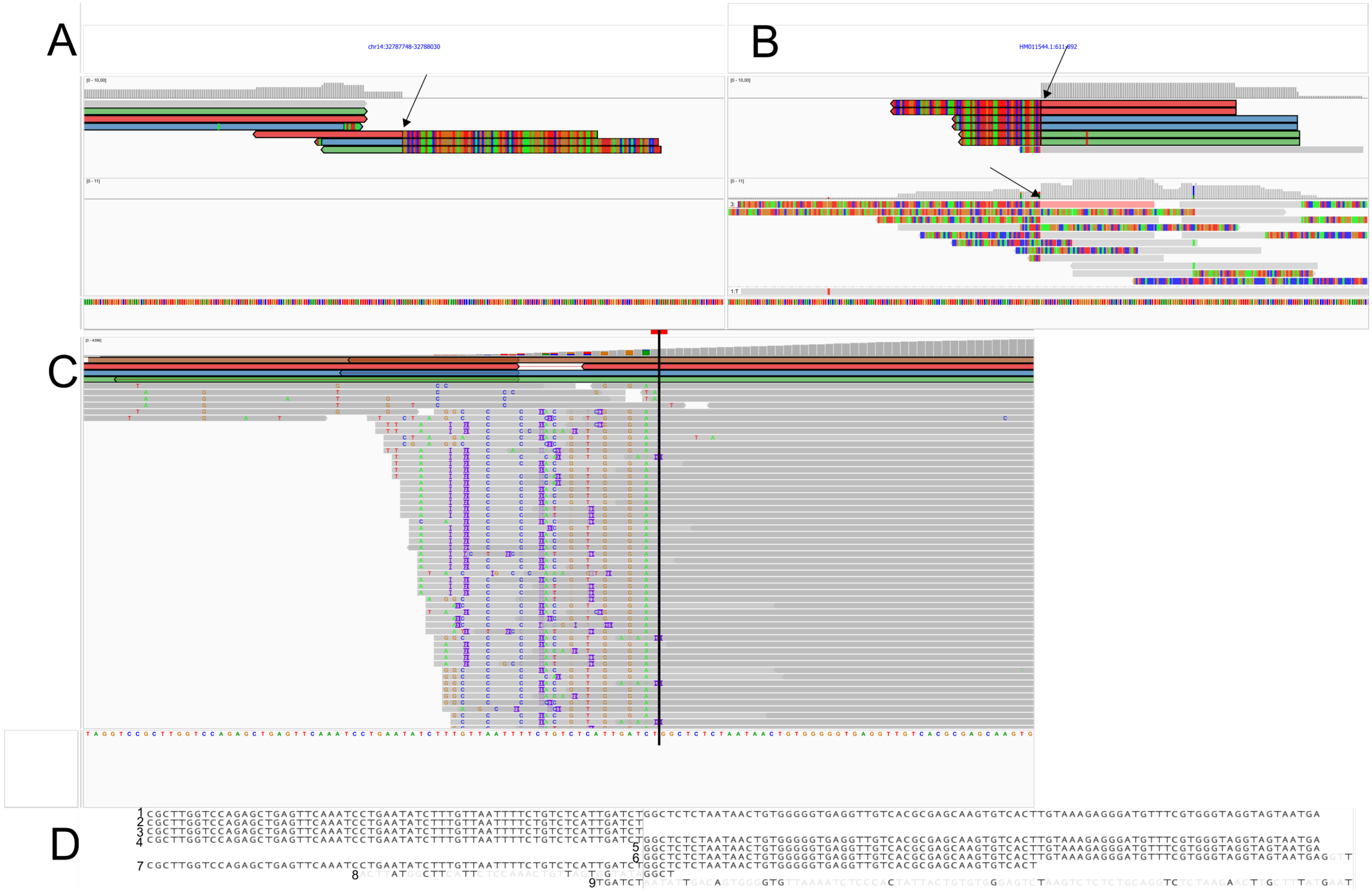

Supplementary Figure S8 Steps of *in silico* integration analysis. *This figure illustrates an example of a junction between MCPyV VP2 and human chromosome 14. The top panels show a split screen of IGV with aligned split reads detected by SurVirus. Vertical colors in split reads represent unaligned sequences (soft-clipped) that map to either virus or human.* ***A)*** *is the view from host side and* ***B****) from the virus side. In* ***B****) the bottom panel shows also aligned denovo contigs with one contig (an arrow and coloring with light red) exhibiting the exact junction area as supported by split reads. In panel* ***C****) are reads realigned with TRACESPipe against a virus-host fusion reference with the junction marked as vertical black line. The reads on the top (with red, blue and green colors) align perfectly to the fusion reference supporting the existence of the junction, while grey reads span from normal human or viral reads. At* ***D****) are alignments of hybrid sequences in descending order: 1) split read sequence aligned to host reference, 2) part of the chimeric sequence that maps to the host reference, 3) host sequence adjacent to the junction as determined by SurVirus, 4) split read sequence aligned to virus reference, 5) part of the chimeric sequence that maps to virus reference, 6) virus sequence adjacent to the junction as determined by SurVirus. 7) Sequence of the denovo contig supporting the junction. 8) and 9) demonstrate how host and virus sequences normally continue without the integration.*

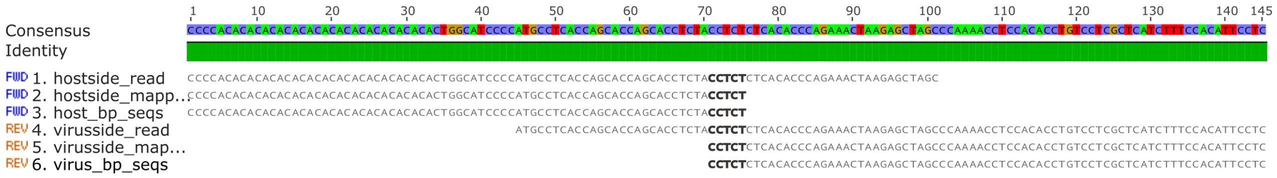

Supplementary Figure S9. *Example of EBV integration in a skin sample. Alignment of two split reads detected by SurVirus, mapping to both human and EBV (first and fourth sequence). Aligned are also parts of those split reads that mapped to the host or virus (second and fifth sequences, respectively) and sequence around the junction based on information on split reads and supporting reads as reported by SurVirus (third and sixth sequence). In this example both EBV and the host shared a common sequence CCTCT (bolded) which supports that integration would have occurred through microhomology-mediated end joining (MMEJ).*

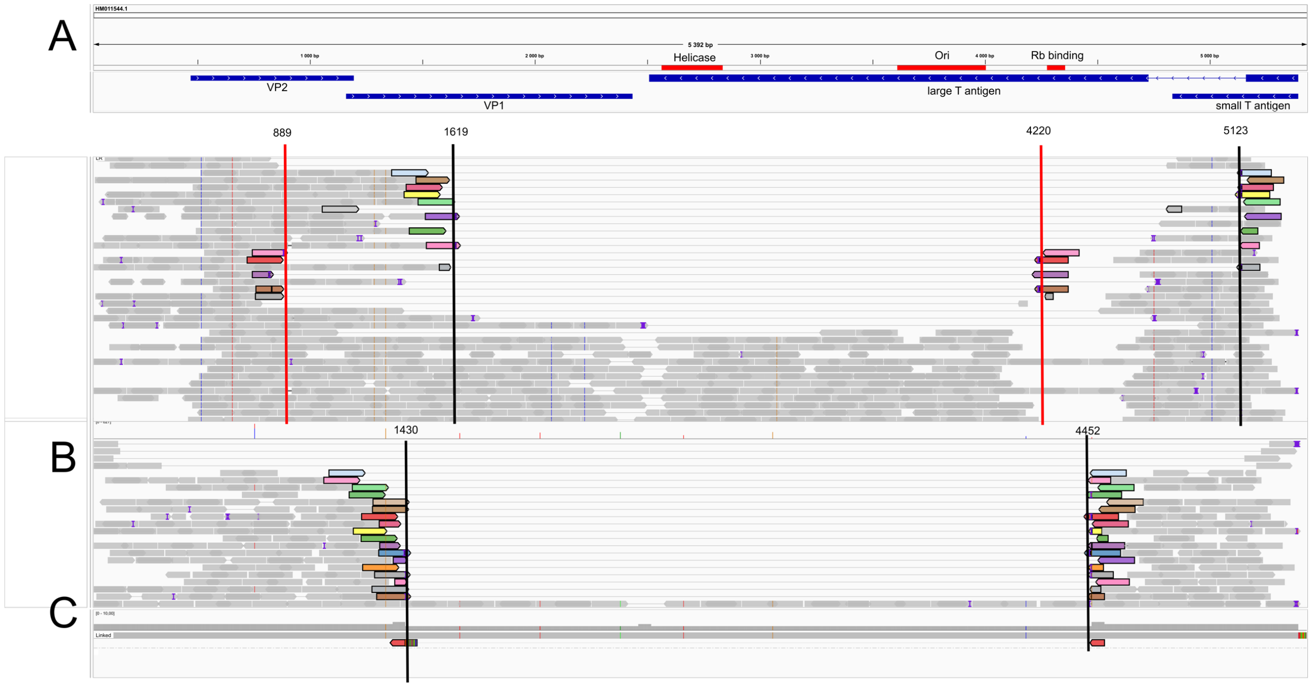

Supplementary Figure S10. Deletions of large T antigen (LT) -gene in Merkel cell polyomaviruses. *Illustrated are two MCPyV genomes with truncations in <1% of the total reads. In the top are MCPyV genome positions and genes. Red markings illustrate different motifs of the LT gene. Read pairs (forward and reverse) are matched with the same color and the gap between the pairs indicate deletion (vertical lines indicate the exact position). In **A)** is MCPyV sequence reads from plucked hair sample that also exhibited MCPyV integration junctions to human genome. Shown here are read pairs evidencing two separate deletions either between large tumor antigen (LT) and VP2 (between red vertical lines) or between small tumor antigen (ST) and VP1 (between black vertical lines). Exact deletion positions were identified by DELLY. The LT-VP2 truncation preserved the RB binding motif of LT gene similarly as seen clonally expanded in Merkel cell carcinomas. In **B)** are MCPyV read pairs from another hair sample exhibiting a deletion between VP1 and LT genes. This truncation introduces a loss of RB binding motif. In **C)** are de novo scaffolds supporting this VP1-LT deletion (reads colored in red). The long contig (in grey) represents the whole MCPyV genome without deletions.*

Supplementary Tables

Supplementary Table S1. Cohort Information.

Individual*	Age	Gender	Underlying cause of death	Manner of death	Reported co(morbidities)	Immunosuppressive / antiviral medication
****Normal core tissue virome**						
2	79	M	traumatic fat embolism	injury	CAD, †atherosclerotic complications, HS	
5	75	M	cerebral laceration	suicide	CAD, atherosclerotic complications, HS	
6	75	M	aortic valve stenosis	disease	CAD, aortic valve stenosis, atherosclerotic complications	
7	74	M	cerebral contusion	injury	CAD, atherosclerotic complications, Parkinson’s disease, type 2 diabetes	
9	73	M	atherosclerotic heart disease	disease	CAD, myocardial infarction, atherosclerotic complications, IgA nephropathy	
11	71	M	atherosclerotic heart disease	disease	atherosclerotic complications	
13	70	M	chronic alcoholism	disease	HS, atherosclerotic complications	
16	68	M	poisoning	injury	CAD, hypertension, atherosclerotic complications	
19	66	M	hypertensive heart disease	disease	hypertension, HS, myocardial hypertrophy and fibrosis	
27	58	M	aortic valve stenosis	disease	CAD, myocardial hypertrophy and fibrosis, atherosclerotic complications	
30	53	F	chronic alcoholism	disease	epilepsy, HS, hepatic cirrhosis,	
**Differing core tissue virome**						
4	77	M	malignant neoplasm of the lung	occupation al disease	Metastatic pulmonary carcinoma in the brain, lymph nodes, liver; CAD, atherosclerotic complications	
10	72	M	drowning	suicide	Relapsed stage IV mantle cell lymphoma, herpes zoster and erythema multiforme 5 weeks before death	rituximab, prednisone, cyclophosphamide, doxorubicin gemcitabine / acyclovir (5 weeks before death)

*Individual numbering corresponds to that of Pyöriä et al. 2023[1] **Normal and differing core tissue virome as determined by virus species and their quantities detected across different organs[1] . † Atherosclerotic complications = glomerulosclerosis / arteriosclerosis obliterans / cerebral atherosclerosis, CAD = coronary artery disease. HS = hepatic steatosis

Supplementary Table S2. All viral genomes and their average coverage breadth and depth

Id	Organ	Breadth	Depth
Parvovirus B19 (B19V)			
13	blood	95 %	5
13	brain	65 %	2
13	colon	98 %	12
13	heart	90 %	4
13	kidney	99 %	9
13	lung	98 %	9
13	skin	100 %	52
11	blood	97 %	14
11	colon	99 %	32
11	kidney	100 %	51
11	liver	51 %	1
11	lung	85 %	4
11	skin	100 %	23
2	blood	76 %	3
2	colon	64 %	2
2	skin	80 %	4
4	colon	100 %	117
4	heart	93 %	4
4	kidney	95 %	11
4	liver	100 %	19
4	lung	73 %	2
4	skin	100 %	82
7	blood	82 %	4
7	colon	72 %	2
7	heart	80 %	3
7	kidney	65 %	2
7	liver	67 %	2
7	lung	94 %	7
7	skin	97 %	7
30	blood	100 %	159
30	brain	92 %	58
30	colon	100 %	729
30	heart	100 %	313
30	kidney	100 %	651
30	liver	78 %	22
30	lung	99 %	9
30	skin	100 %	376
5	brain	79 %	3
5	colon	100 %	23
5	heart	71 %	2
5	kidney	91 %	4
5	liver	100 %	73
5	lung	86 %	6
5	skin	100 %	59
9	colon	94 %	5
9	lung	100 %	35
9	skin	100 %	37
Id	Organ	Breadth	Depth
16	colon	92 %	7
16	heart	88 %	3
16	kidney	100 %	25
16	skin	100 %	90
19	colon	82 %	3
19	kidney	100 %	7
19	lung	100 %	45
10	brain	82 %	2
10	colon	89 %	3
10	heart	52 %	1
10	kidney	96 %	9
10	lung	62 %	2
10	skinfem*	100 %	88
Herpes Simplex 1 virus (HSV-1)			
2	hair	65 %	3
4	blood	91 %	6
4	colon	57 %	4
4	lung	52 %	7
Varicella zoster virus (VZV)			
10	skin	98 %	32
Epstein-Barr virus (EBV)			
4	hair	55 %	3
10	skin	97 %	47
Human cytomegalovirus (HCMV)			
4	heart	66 %	3
4	liver	53 %	2
4	lung	69 %	5
4	skin	56 %	3
Human herpesvirus 6B (HHV-6B)			
13	colon	94 %	19
11	colon	79 %	5
11	kidney	98 %	88
11	liver	93 %	17
11	lung	73 %	3
6	kidney	95 %	32
6	liver	93 %	5
7	blood	81 %	5
7	colon	69 %	2
7	liver	98 %	58
7	lung	56 %	2
30	kidney	73 %	3
30	liver	70 %	1
5	liver	80 %	5
5	hair	75 %	3
27	kidney	97 %	29
27	liver	100 %	41
Id	Organ	Breadth	Depth
9	colon	94 %	9
9	kidney	89 %	10
9	colon	96 %	9
9	kidney	99 %	68
9	lung	61 %	2
Human herpesvirus 7 (HHV-7)			
13	colon	98 %	6
11	colon	95 %	1
11	liver	46 %	1
4	liver	91 %	3
4	colon	47 %	1
7	colon	96 %	3
16	colon	67 %	1
19	colon	86 %	3
JC Polyomavirus (JCPyV)			
11	heart	68 %	2
2	kidney	98 %	22
2	hair	85 %	4
4	blood	97 %	5
4	colon	92 %	5
4	heart	54 %	1
4	kidney	100 %	140
4	liver	99 %	39
4	lung	90 %	5
6	kidney	81 %	2
6	liver	54 %	1
7	blood	86 %	5
7	kidney	100 %	42
7	hair	60 %	2
Merkel Cell polyomavirus (MCPyV)			
11	hair	100 %	197
11	colon	58 %	2
2	hair	100 %	76
7	colon	66 %	2
7	skin	79 %	4
7	hair	100 %	12 227
10	hair	100 %	732
10	skin	58 %	1
5	hair	99 %	45
9	hair	100 %	147
16	hair	72 %	2
HPyV6			
9	hair	97 %	8
HPyV7			
5	hair	79 %	2
HPV			
2	hair	74 %	238

*Skin sample taken from right femoral area. Other skin sample from the same individual was taken from right facial area.

Supplementary Texts

Supplementary Text S1. Detailed description of MCPyV minor variants (MV)

The VP2 and LT genes of MCPyV contained the majority of MVs (n=23 and 19, respectively). The mean frequency of alternative nucleotides at MV positions was 17% (Supplementary Figure S2B), and 7 % of MV positions were shared between at least two samples. MCPyV iSNVs were enriched in A to G transversions compared to G to A (p=0.04, two-sided binomial test), (Supplementary Figure S3B). No MCPyV MVs were identified in the skin or colon, although these samples had greatly lower sequencing breadth and depth (average 65% and 3X, respectively).

Supplementary Text S2. Detailed description of HHV-6B intra-host variability

We identified inter-tissue differences in HHV-6B in five individuals: one displayed 10 variations in the HHV-6B genome between liver and hair, while the other four exhibited 1-4 variations across organs. These differences consisted of 10 cSNVs and nine indels, of which seven indels were seen in short homopolymer regions. These differences affected nine genes across HHV-6B genome of which five have been confirmed as an antigen for HHV-6B specific CD4+ or CD8+ T cells (Table 1, Figure 4A), [2].

Out of total 15 MVs seen in HHV-6B genomes, eight were iSNVs of which five within genes. These consisted of one non-synonymous mutation in U47, three non-synonymous mutations in U86 and one synonymous mutation in U51 (Table 1, Figure 4B). U47 and U86 have been implicated as targets of HHV-6B specific CD4+ or CD8+ T cells, respectively [2]. Furthermore, altogether four MVs were located in introns of U77 and U100. The seven MV indels detected in HHV-6B were all located in microsatellite areas containing 9-13

homopolymer repeats of T, G or A. MV indels were identified by both variant callers iVar and LoFreq. Analysis with GangSTR supported that the genomes contained two versions of a tandem repeat in these positions (Supplementary Dataset S2). The mean minor nucleotide frequency was 24% (Supplementary Figure S2C). 19% of MV positions were shared with by least one other sample.

Supplementary Text S3. Detailed description of HHV-7 intra-host variability

We performed an intra-host comparison of HHV-7 consensus sequences retrieved from colon and liver samples of two individuals (Table 1, Supplementary Figure S5A, Supplementary Dataset S1). In one subject, we detected two consensus mutations, and in another, three. Out of these 5 mutations, four were cSNVs. Three consensus differences were observed in the major capsid protein (U57), one in the large tegument protein (U31) and one in the glycoprotein H (U48). Although, data on specific T cell epitopes for HHV-7 are lacking, studies on the closely related HHV-6B have shown that all the mentioned protein homologs are targeted by respective HHV-6B CD4+ T cells [2].

M and π were higher in non-CDS than in CDS, although lacking statistical significance (M, p=1.00, permutation test of means), (Figure 1A, C, Supplementary Figure S1A).

With regards to minor variants, we identified one non-synonymous iSNV in U30 and another iSNV in U100 intron area. (Supplementary Figure S5B, Table 1). Similarly to consensus-level variation, the PS and H were also higher in non-CDS, yet not statistically significant (PS, p=1.00; H, p=0.67, permutation test of means), (Figure 1B, C, Supplementary Figure S1B, Supplementary Dataset S2).

Supplementary Text S4. Detailed description of minor variants in EBV

EBV genome reconstructed from a skin sample of an individual with relapsed mantle cell lymphoma contained the highest amount of MVs (51) in a single sample from all the virus genomes in this cohort. In total 47 iSNVs and 4 indels were detected. MVs were seen across the genome in 20 genes, particularly in latency proteins EBNA-2 (n=5) and EBNA-1 (n=4), which are common targets for EBV specific CD4+ and CD8+ cells (Table 1), [3,4]. From the four indels, three were within homopolymer microsatellites (Two containing C repeat and one containing G repeat with lengths of 9 to 11) that GangSTR confirmed to contain two versions of the tandem repeat (Supplementary Dataset S2).

Supplementary Text S5. Detailed description of intra-host diversity of HCMV and HSV-1

One individual with metastatic pulmonary carcinoma exhibited reactivation of HSV-1 and HCMV across the organs. We compared HCMV consensus sequences obtained from this subject's heart, liver, lung, and skin. Specifically, we identified ten cSNVs and eight indels of which four were in homopolymer repeats (Figure 6A, Table 1, Supplementary Dataset S1). Within genes, most mutations were concentrated to UL112 (n=3) or UL 44 (n=2) which are frequently recognized by HCMV specific CD4+ and CD8+ T cells [5,6].

Minor variants of HCMV were detected in all 4 tissues of this individual. Similarly, to other viruses, non-CDS exhibited higher diversity among MVs (PS, p=0.03; H, p=0.15, permutation test of means), (Figure 1B, C, Supplementary Figure S1B, Supplementary Dataset S2).

HCMV MVs consisted of nine iSNVs and ten indels. Within genes, MVs occurred most frequently in UL42 (n=5), (Figure 6B, Table 1). 18 % of MV positions were identical at least between two samples.

We compared the inter-tissue variation of HSV-1 between this individual's blood, colon and lung. We found altogether five indels (Supplementary Figure S6A, Table 1, Supplementary Dataset S1) of which four were in short homopolymer repeats.

Furthermore, we observed eight and four HSV-1 MVs in colon and blood samples of this individual, respectively. Of these, eleven were iSNVs and one was indel (Table 1, Supplementary Figure S6, Supplementary Dataset S2). Only one iSNV was within a gene (US6). Thus, HSV-1 MVs were more frequent in non-CDS regions, although lacking significance (PS, p=0.40; H, p=0.10, permutation test of means), (Figure 1B, C, Supplementary Figure S1B).

Supplementary references

1. Pyöriä,L., Pratas,D., Toppinen,M., Hedman,K., Sajantila,A. and Perdomo,M.F. (2023) Unmasking the tissue-resident eukaryotic DNA virome in humans. Nucleic Acids Res, 51, 3223–3239.
2. Hanson,D.J., Hill,J.A. and Koelle,D.M. (2018) Advances in the characterization of the T-cell response to human herpesvirus-6. Front Immunol, 9, 376651.
3. Taylor, G. S., Long, H. M., Brooks, J. M., Rickinson, A. B. & Hislop, A. D. The immunology of epstein-barr virus-induced disease. *Annu Rev Immunol* **33**, 787–821 (2015).
4. Cohen,J.I. (2024) Therapeutic vaccines for herpesviruses. J Clin Invest, 134, e179483.
5. Sylwester,A.W., Mitchell,B.L., Edgar,J.B., Taormina,C., Pelte,C., Ruchti,F., Sleath,P.R., Grabstein,K.H., Hosken,N.A., Kern,F., et al. (2005) Broadly targeted human cytomegalovirus-specific CD4+ and CD8+ T cells dominate the memory compartments of exposed subjects. Journal of Experimental Medicine, 202, 673–685.
6. Jackson,S.E., Sedikides,G.X., Mason,G.M., Okecha,G. and Wills,M.R. (2017) Human Cytomegalovirus (HCMV)-Specific CD4 + T Cells Are Polyfunctional and Can Respond to HCMV-Infected Dendritic Cells In Vitro. J Virol, 91, 2128–2144.

Supplementary datasets

Supplementary Dataset S1: Intra-host consensus sequence differences detected between tissues

Supplementary Dataset S2: Minor variants detected within each sample

Supplementary Dataset S3: All integration junctions identified with by SurVirus
